# The effect of exercise and nutrition interventions on physical functioning in patients undergoing haematopoietic stem cell transplantation: a systematic review and meta-analysis

**DOI:** 10.1007/s00520-021-06334-2

**Published:** 2021-06-16

**Authors:** Marianne C. Prins, Gerben van Hinte, Niek Koenders, Anne Lieke Rondel, Nicole M. A. Blijlevens, Manon G. A. van den Berg

**Affiliations:** 1 Radboud University, Nijmegen, The Netherlands; 2 Department of Rehabilitation, Radboud Institute for Health Sciences, Radboud University Medical Centre, Nijmegen, The Netherlands; 3 Department of Gastro Enterology and Hepatology-Dietetics, Radboud Institute for Health Sciences, Radboud University Medical Centre, HP 459, Po Box 9101, 6500 HB Nijmegen, The Netherlands; 4 Department of Hematopoietic Diseases, Radboud University Medical Centre, Nijmegen, The Netherlands

**Keywords:** Exercise, Nutrition, Stem cell transplantation, Physical functioning, Systematic review

## Abstract

**Purpose:**

Haematopoietic stem cell transplantation (HSCT) is potentially lifesaving. However, it comes with negative consequences such as impaired physical functioning, fatigue and poor quality of life. The aim of this systematic review and meta-analysis is to determine the effect of exercise and nutrition interventions to counteract negative consequences of treatment and improve physical functioning in patients receiving HSCT.

**Methods:**

This systematic review and meta-analysis included randomised controlled trials from three electronic databases between 2009 and 2020. The trials included adult patients receiving HSCT and an exercise or nutrition intervention. Study selection, bias assessment and data extraction were independently performed by two reviewers. Physical functioning outcomes were meta-analysed with a random-effects model.

**Results:**

Thirteen studies were included using exercise interventions (*n* = 11) and nutrition interventions (*n* = 2); no study used a combined intervention. Meta-analysis of the trials using exercise intervention showed statistically significant effects on 6-min walking distance (standardised mean difference (SMD) 0.41, 95% CI: 0.14–0.68), lower extremity strength (SMD 0.37, 95% CI 0.12–0.62) and global quality of life (SMD 0.27, 95% CI: 0.08–0.46).

**Conclusion:**

Our physical functioning outcomes indicate positive effects of exercise interventions for patients receiving HSCT. Heterogeneity of the exercise interventions and absence of high-quality nutrition studies call for new studies comparing different types of exercise studies and high quality studies on nutrition in patients with HSCT.

**Supplementary Information:**

The online version contains supplementary material available at 10.1007/s00520-021-06334-2.

## Introduction

Haematopoietic stem cell transplantation (HSCT) is a potentially lifesaving treatment for many hematologic malignancies, such as multiple myeloma, lymphoma and leukaemia. Annually about 70,000 HSCT’s are performed worldwide [1]. The number of patients undergoing HSCT has rapidly increased over the past decades and has not shown any signs of plateauing so far [2]. Long-term survival rates have been rising, due to advancement in the management of complications [3]. However, despite saving many life years, this treatment often comes with complications, such as graft-versus-host disease (GvHD), infections and oral mucositis, that lead to life years lived with disabilities and transplant-related mortality [4, 5].

Apart from these complications, many post-HSCT patients suffer from negative consequences of treatment such as impaired physical functioning, a decreased quality of life (QOL) and fatigue [6–9]. Adding exercise interventions to usual care might contribute to the improvement of physical functioning, fatigue and QOL in haematopoietic stem cell recipients [10–12]. A 2019 Cochrane review shows that there is moderate- to low-quality evidence that aerobic physical exercise positively influences physical functioning in adult patients with and without HSCT for haematological malignancies [13]. However, a 2018 meta-analysis by Liang et al. showed no statistically significant effects of exercise interventions on cardiorespiratory fitness and upper muscle strength in HSCT patients. It did show statistically significant positive effects on lower muscle strength, fatigue and QOL [14].

In addition, the effects of nutritional interventions for HSCT recipients are studied in two other reviews, showing inconclusive evidence [15, 16]. In patients undergoing chemo- or radiotherapy, nutritional interventions seem to have a positive effect on preservation of nutritional status and QOL [17, 18]. The merits of nutritional interventions for physical functioning were not clear [18].

Cancer rehabilitation is incrementally seen as an intricate part of a patient’s medical treatment [19]. Patients are motivated to improve their health through lifestyle changes after diagnoses of cancer to prepare for the intensive upcoming treatment, serving as an opportunity to increase physical functioning in HSCT recipients through exercise and nutrition interventions [20]. Since the publication of the Cochrane review, several new studies with exercise interventions have been published [13]. On top of that, recent systematic review on nutrition does not solely include randomised clinical trials, which limits our confidence in the estimation of true intervention effects [15]. Hence, the aim of this study is to determine the effect of exercise and nutrition intervention on physical functioning in patients receiving haematopoietic stem cell transplantation. Secondary outcome are QOL, fatigue, body fat, weight, length of stay (LOS), mortality and GvHD.

## Materials and methods

The PRISMA (Preferred Reporting Items for Systematic Reviews and Meta-Analyses) statement was followed [21]. A completed PRISMA checklist is provided in Supplementary Appendix A.

### Eligibility criteria

This systematic review and meta-analysis includes randomised controlled trials (RTC’s) that were published between 1 January 2010 and 1 July 2020.

Studies that included adults (16 years and older) who are planned for or have received autologous or allogenic HSCT were included. Only studies in which at least 75% of patients undergo HSCT with at least ninety percent of patients suffering from a haematological malignancy were included. Studies that are not available in full text or English were excluded.

Studies with exercise and nutrition interventions before, during or after the transplant were included. Only exercise studies that involve aerobic, strength or whole body vibration training were included. Exercise interventions based on inspiratory muscle training, yoga, tai-chi and chi-gong were excluded. Studies that include vigorous exercise in the treatment plan of patients in the control group were excluded. Studies with nutrition interventions that aimed towards reaching a calorie and/or protein goal were included.

### Information sources and search

Three electronic databases were searched by MP, in specific PubMed, Embase and the Cochrane Central Register of Controlled Trials (CENTRAL). A search strategy was designed with the help of an information specialist (OYC) and included thesaurus terms and free text terms on the population and the intervention. Search terms on the outcomes were not included to maximise the sensitivity of the search. For the detailed search strategy, see Supplementary Appendix B. A randomised-controlled trial filter designed by the Cochrane collaboration was added in PubMed and Embase [22]. In Embase, conference abstracts were excluded. References of all included trials and relevant reviews were checked by MP for further literature.

### Study selection

Two independent researchers (MP and GH) performed the title abstract screening using Rayyan [23]. In accordance with the PRISMA statement, reasons for exclusion in the full-text screening were documented [21]. Disagreement between the reviewers was resolved by discussion and mutual agreement. Cohen’s *κ* was used to assess agreement between the reviewers.

### Data collection process

One reviewer (MP) extracted all relevant data, which was checked by a second reviewer (GH). In accordance with the guidelines proposed by the Cochrane Handbook, an adapted Cochrane Data Collection Form was used to extract data [22]. Collected data included a summary of the following: (1) study design and general information, (2) participant data, (3) intervention details, (4) outcome measures and (5) results. If different studies reported outcomes of the same patient population, data were combined into one form. If studies did not provide sufficient data for meta-analysis, authors were contacted.

### Outcomes

The primary outcome in this study is physical functioning. All outcomes for aerobic capacity (e.g. 6-min walking distance and peak oxygen consumption), strength (e.g. hand grip strength, lower- and upper muscle strength) and functional performance were considered. Secondary outcome measures were as follows: QOL, fatigue, body fat, weight, length of hospital stay (LOS), mortality and GvHD.

### Risk of bias

Two independent reviewers (MP and GH) assessed the risk of bias using the Cochrane Risk of Bias tool 2.0 for the following five domains: (1) bias arising from the randomization process, (2) bias due to deviations from intended interventions, (3) bias due to missing outcome data, (4) bias in measurement of the outcome and (5) bias in selection of the reported result. The risk of bias domains was scored as ‘low risk,’ ‘high risk’ or ‘some concern.’ Discrepancies were discussed and resolved through mutual agreement. A third author (MB) was consulted if necessary.

### Data synthesis and analysis

Meta-analyses were performed with Review Manager 5.4 [24]. The meta-analysis was performed if at least three studies had measured the same outcome. Data from the first time point post-intervention were selected. Nutrition and exercise studies were considered for meta-analysis separately, due to the heterogeneity of the interventions. When various studies used different scales for continuous outcomes, standardised mean differences (SMD) were calculated; otherwise, mean differences (MD) were calculated. Heterogeneity of the treatment effects between trials was assessed using the *I*^2^ test, with *I*^2^ = 50–90% indicating substantial heterogeneity and *I*^2^ = 75–100% indicating considerable heterogeneity [25]. Because considerable heterogeneity was expected, a random effects model was used. Differences of 0.2, 0.5 and 0.8 standard deviations were considered as ‘small,’ ‘medium’ and ‘large’ effect sizes respectively [26].

## Results

**Fig. 1:**
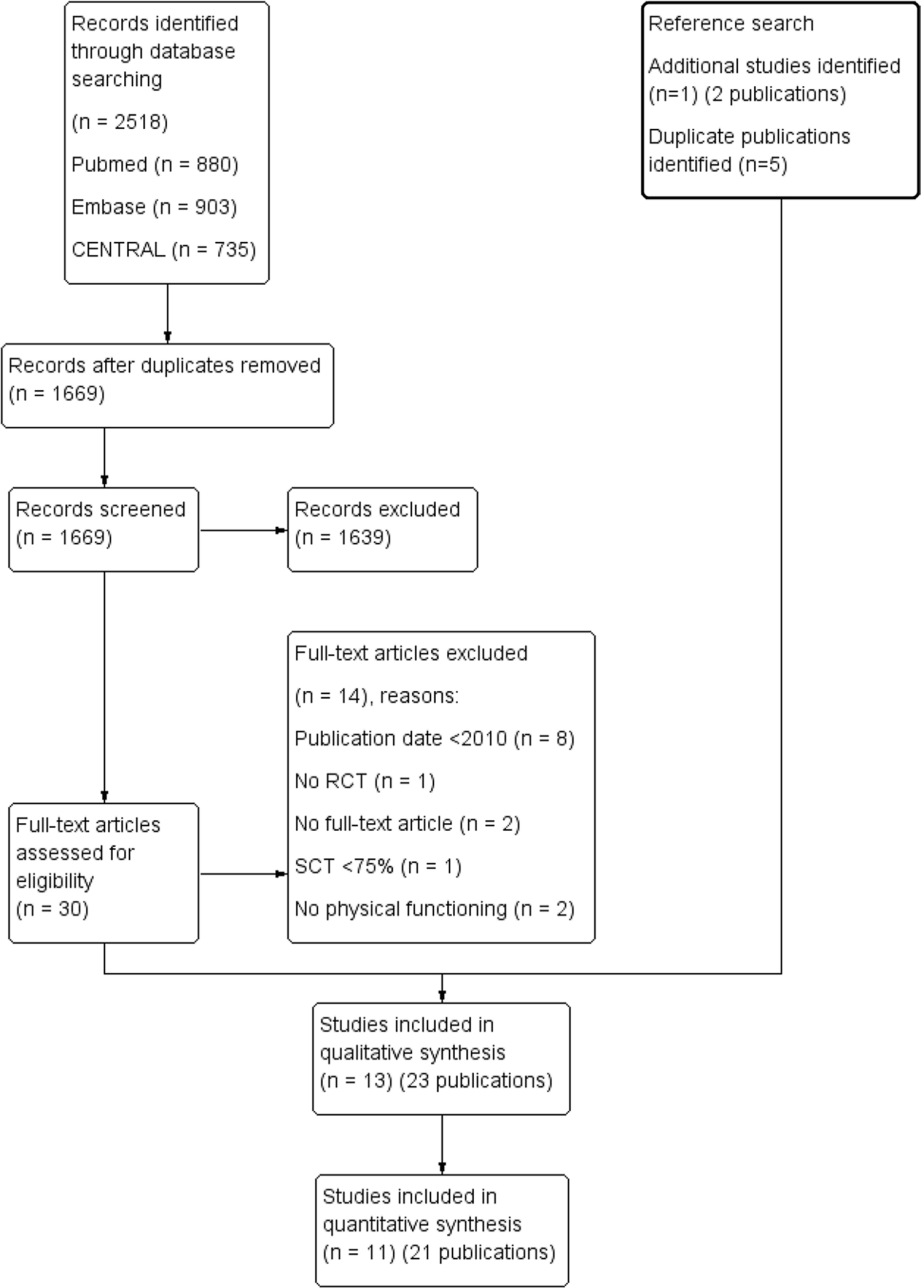
Flow diagram

The process of study inclusion and the reasons for exclusion are presented in an adapted Preferred Reporting Items for Systematic Reviews and Meta-Analyses (PRISMA) flow diagram (Fig. 1). The searches acquired in total 2518 records. After duplicate removal, 1669 records underwent title abstract screening in Rayyan. Thirty records were screened for full text, of which fourteen were excluded. Twelve studies (sixteen publications) met the inclusion criteria and were forward and backward reference searched, which identified one additional study (two publications) and five other duplicate publications. In total, thirteen studies with 23 publications were included in this review [27–49]. Eleven studies (21 publications) were included in the meta-analysis. Agreement of study inclusion between the two reviewers was almost perfect (kappa = 0.90, 95% CI 0.76–1).

### Study characteristics

The thirteen included studies comprised data of 954 patients (range 19–187 patients). An overview of the study populations is shown in Table 1. Mean age ranged from 30 to 63. In all studies, over half of the population was male. HSCT type and diagnosis varied widely across studies, including multiple myeloma and numerous types of leukaemia and lymphoma. Eleven studies included an exercise intervention and two studies included nutrition interventions.

**Table 1:** Study characteristics

Study	Country	No. of patients randomized/baseline/follow-up	Mean age (range)	No (%) of men	Auto-SCT/allo-SCT	Diagnosis (*n*)	Intervention type
Baumann 2010 [27, 28]	Germany	64/64/49	44.5^a^ (?)	35 (54.7)	18/46	ALL (9); AML (25); CML (3); MDS/MPS (6); MM (9); NHL/CLL (8); solid tumour (3); variable immunodeficiency (1)	Exercise
Coleman 2012 [29–32]	USA	187/187/166	56.2 (25–76)	109 (58.3)	187/0	MM (187)	Exercise
Hacker 2011 [33]	USA	19/19/17	46.3 (20–67)	14 (73.6)	13/6	Unknown (19)	Exercise
Hacker 2017 [34, 35]	USA	75/67/67	53.3 (19–73)	41 (61.2)	39/28	ALL (4); AML (14); CLL (2); CML (3); HL (2); MDS (6); MM (28); NHL (8)	Exercise
Jabbour 2019 [36]	Libanon	52/46/46	45.2 (25–57)	30 (65.2)	29/17	AML (12); Ewing sarcoma (1); lymphoma (25); MM (8)	Nutrition
Knols 2011 [37]	Switzerland	131/131/114	46.7 (18–75)	77 (58.8)	80/51	ALL (2); AML (31); amyloidosis (1); CLL (14); HL (14); NHL (25); MM (37); osteomyelofibrosis (4); testicular cancer (3)	Exercise
Koutoukidis 2020 [38, 39]	UK	131/93/85	63 (35–86)	51 (54.8)	82/11	MM (93)	Exercise
Pahl 2020 [40]	Germany	71/44/44	55.6 (32–63)	30 (68.2)	0/44	ALL (3); AML (26); CLL (1); CMML (1); common variable immunodeficiency (1); Lymphoma (4); MDS (3); MM (1); myelofibrosis (1); SAA (2); septic granulomatosis (1)	Exercise
Persoon 2017 [41–44]	Netherlands	109/109/97	54.8^a^ (19–67)	69 (63.3)	109/0	MM (58); (N)HL (51)	Exercise
Ren 2017 [45]	China	50/24/24	30.4 (?)	16 (66.7)	0/24	ALL (11); AML (13)	Nutrition
Santa Mina 2020 [46]	Canada	30/30/12	49.4 (?)	15 (50.0)	0/30	Leukemia (21); lymphoma (1); MDS (6); MNGIE (1)	Exercise
Wiskemann 2011 [47, 48]	Germany	112/112/80	48.8 (18–71)	71 (67.6)	0/105	AA (2); ALL (14); AML (22); CML (4); CLL (4); MDS (12); MM (3); MPS (13); other lymphomas (20); secondary AML (11)	Exercise
Wood 2020 [49]	USA	34/28/16	52^a^ (28–73)	16 (57.1)	0/28	AA (1); ALL (3); AML (15); CML (1); HL (1); HLH (1); mantle cell lymphoma (1); MDS (3); MM (1); myelofibrosis (1)	Exercise

^a^Median. *Auto-SCT* autologous HSCT, *Allo-SCT* allogenic HSCT, (*S)AA* (severe) aplastic anemia, *ALL* acute lymphoblastic leukemia, *AML* acute myelogenous leukemia, *CLL* chronic lymphoblastic leukemia, *CML* chronic myelogenous leukemia, *CMML* chronic myelo-monocytic leukemia, *HL* Hodgkin lymphoma, *HLH* hemophagocytic lymphohistiocytosis, *MDS* myelodysplastic syndrome, *MM* multiple myeloma, *MNGIE* mitochondrial neurogastrointestinal encephalomyopathy, *MPS* myeloproliferative syndrome, *NHL* non-Hodgkin lymphoma

**Fig. 2:**
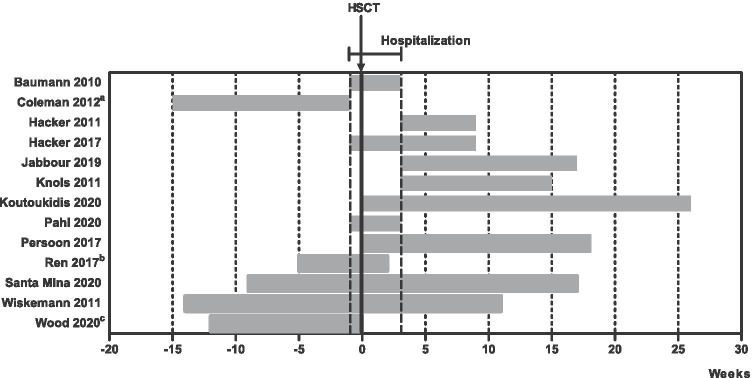
Intervention timing and duration. **a** HSCT planned for outpatient setting. **b** “From enrolment to post-transplantation.” **c** Five- to twelve-week pre-HSCT

Table 2 shows the interventions. In eight studies, the frequency of exercise intervention ranged from two to four times a week [29, 33, 34, 37, 38, 46, 47, 49] and two studies consisted of daily exercise intervention. [27, 40]. Eleven studies included an exercise intervention and two studies included nutrition interventions. Two exercise intervention studies only included aerobic exercise [27, 49], three only included strength training [33–35, 40] and the remaining six studies combined both aerobic exercise and strength training [32, 37–39, 41–44, 46–48]. Three out of the 11 exercise studies were entirely unsupervised [29–32, 47–49]. The duration of the interventions varied widely from about 30 days to 6 months. They took place before (*n* = 5)[29–32, 45–49], during (*n* = 9) [27–32, 34, 35, 38–48] and after (*n* = 8)[33, 34, 36–39, 41–44, 46–48] the hospitalisation period (Fig. 2). The adherence of patients in the intervention group ranged from 20 to 97%.

**Table 2:** Intervention characteristics

	Excercise intervention				Nutrition			
Study	Frequency	Intensity	Type	Timing	Nutrition	Intervention group	Supervision	Usual care group
Baumann 2010 [27]	Daily	80% of watt max	Aerobic and	During hospitalization		Aerobic exercise: 10–20 min on a bicycle ergometer at 80% of achieved watt load in a WHO-endurance test. During aplasia twice daily, after engraftment once daily	Yes	Supervised low intensity passive and active mobilization consisting of gymnastics, massages, extensions and coordination training, 5 days a week
			ADL training			Other components: ADL training consisting of walking, stepping and stretching, 20 min daily		
Coleman 2012 [29]	3 to 4 times a week	65–80% max. heart rate	Aarobic	Before hospitalization		Aerobic exercise: walking at 65–80% of maximum heart rate, 3–4 days a week	No	Recommendation to walk 20 min three times a week
		80% of 1RM	Strength			Strength training: training extremities at 80% of 1RM, 3–4 days a week		
						Other components: stretching exercises for hamstrings, shoulder rotation, calves, hip flexors and triceps; daily		
Hacker 2011 [33]	3 times a week	Goal: Borg scale 13	Strength	After hospitalization		Strength training: progressive resistance training using elastic resistance bands and body weight for resistance, 3 times a week. Intensity somewhat hard (Borg scale 13)	Partly	Recommendations regarding rest, physical activity and exercise from HSCT physician
Hacker 2017 [34]	3 times a week	Goal: Borg scale 13	Strength	During and after		Strength training: progressive resistance using elastic resistance bands and body weight for resistance. Intensity somewhat hard (Borge scale 13, 3 times a week)	Partly	Two visits a week to discuss hospital experience during hospitalization. Standardized 1-on-1 education intervention, once a week after discharge, for 6 weeks
				hospitalization		Other components: active range of motion exercises; as many as possible, 2 times a week		
Jabbour 2019 [36]					30–35 kcal and 1.5 g protein	Main intervention: advise on a diet high in energy and protein: 30–35 cal and 1.5 g of protein per kg adjusted weight, once a month	n/a	Advise on food safety guidelines at discharge
					Per kg adjusted weight	Other components: advise on food safety guidelines. Encouragement to exercise: 150 min of moderate intensity activity through-out the week and muscle-Strengthening activities 2 or more days a week		
Knols 2011 [37]	2 times a week	50–80% max. heart rate	Aerobic	After hospitalization		Aerobic exercise: ergometer-cycling: 50–80% of estimated maximum heart rate, 2 times a week	Yes	Usual care
			Strength			Strength training: progressive resistance training with dumbbells		
Koutoukidis 2020 [38]	3 times a week	50–75% max. heart rate	Aerobic	During and after hospitalization		Aerobic exercise: training on treadmill walking, cycle ergometer, cross-trainer or stepper at 50–75% of predicted maximum heart rate, 3 times a week	Partly	Asked to maintain usual lifestyle
		max. 10 repetions	Strength			Strength training: resistance exercise using weightlifting equipment, body weight or resistance bands: progressed by 10 repetition maximum		
Pahl 2020 [40]	Daily	Goal: Borg scale 14–16	Strength	During hospitalization		Strength training: whole body vibration training of the legs, 20 min daily. Intensity goal of Borg scale 14 to16	Yes	Supervised mobilization of the spine and stretching of the whole body sitting or lying in bed or standing in front of it for 20 min daily
Persoon 2017 [41]	2 times a week (13 weeks);	30–65% of MSEC	Aerobic	During and after hospitalization		Aerobic exercise: interval training on a bicycle ergometer: 2 × 8 min at 30–65% of MSEC	Yes	Not specifically motivated to exercise, but not restricted
	Later 1 time a week	65–80% of 1RM;	Strength			Strength training: resistance exercises 10 per set at 65–80% of 1RM, later 20 per set at 35–40% of 1RM. Twice weekly, from week 13 onwards once weekly		
		Later 35–40% of 1RM				Other components: five motivating counseling sessions		
Ren 2020					1.5 g/kg/day and	Main intervention: ingestion of 1.5 g/kg/day blended protein (50% whey, 50% soy) per day	n/a	Given standardized dietary recommendations: 35 kcal/kg/d and 1,5 g/kg/d protein
					35 kcal/kg/day	Other components: given standardized dietary recommendations: 35 kcal/kg/d and 1,5 g/kg/d protein		
Santa Mina 2020 [46]	3 times a week	60–80% of heart rate	Aerobic	Before, during and		Aerobic exercise: training using stationary cycle, treadmill, elliptical machine or briskly walking at 60–80% of heart rate reserve for 10–15 min	Partly	Standard physiotherapy to maintain ability to perform activities of daily living. At 100 days post-discharge offered the same program as rehabilitation in intervention group
		Individually modified to maintain	Strength	after hospitalization		Strength training: resistance training using free weights and/or resistance bands. Prehabilitation and		
		Sufficient training stimulus				posthabilitation for 30–45 min, inpatient 10–30 min. Both three times a week		
Wiskemann 2011 [47, 48]	3 times a week	Goal: Borg scale 12–14	Aerobic	Before, during and		Aerobic exercise: endurance training: (Nordic) walking, bicycling, jogging for 15–40 min at Borg scale 12–14. Three times a week	No	Physiotherapy offered up to 3 sessions a week during hospitalization (standard care)
	2 times a week	Goal: Borg scale 14–16	Strength	after hospitalization		Strength training: exercises for the upper and lower extremities with and without stretch bands: 8–20 reps, 2–3 sets: at Borg scale 14–16. Two times a week		
						Other components: weekly phone calls for questions and to review adherence		
Wood 2020 [49]	3 to 4 times a week	80% max. heart rate	Aerobic	Before hospitalization untill SCT		Aerobic training: interval training: walking, jogging, running, cycling, elliptical or stair climbing, 5 min warm up followed by five 2-min intervals targeting 80% of maximum heart rate and 3-min low-intensity recovery intervals. 3–4 times a week	No	Weekly scripted calls, not provided with motivational message. Wore Fitbit
						Other components: individual counseling, weekly motivational phone calls and Fitbit step reminders		

*ADL* activities of daily living, *n/a* not applicable, *1RM* one repetition maximum, *MSEC* maximal short excercise capacity, *SCT* stem cell transplantation

### Risk of bias

**Fig. 3:**
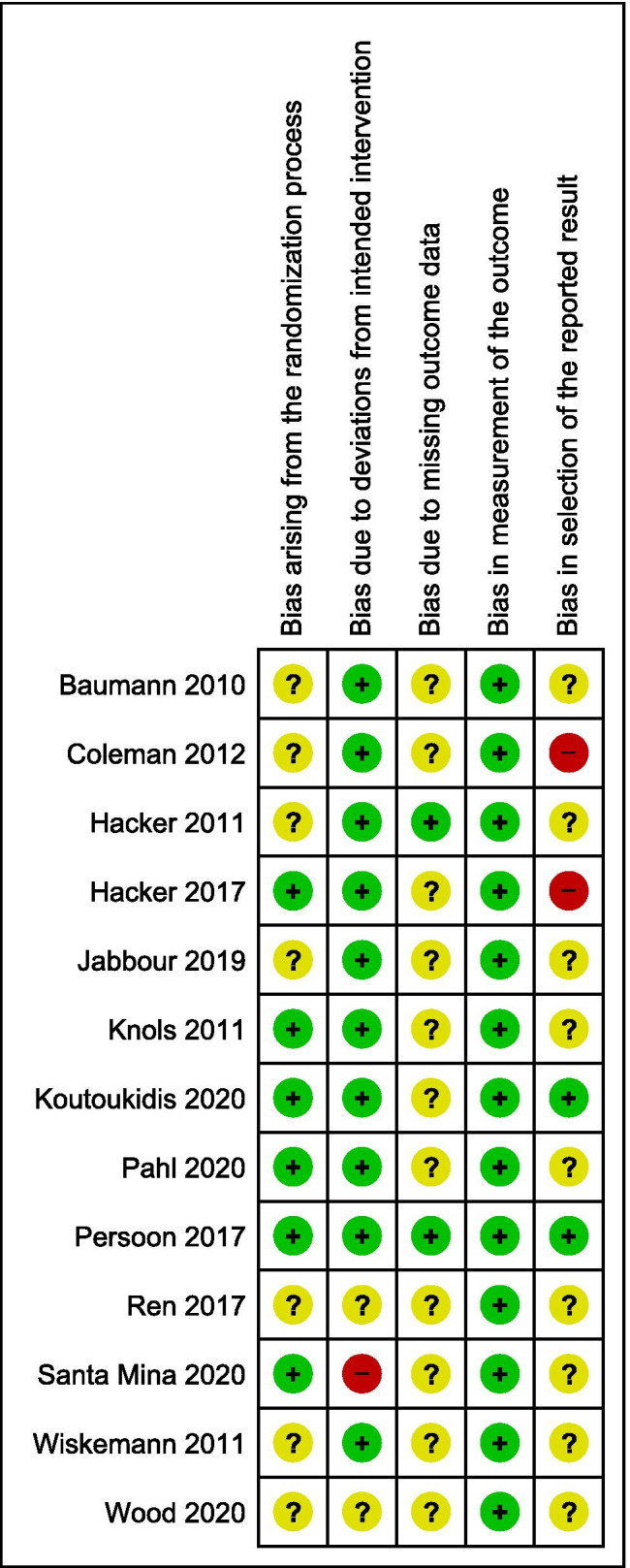
Risk of bias summary: review authors' judgements about each risk of bias item for each included study

An overview of the results of the bias assessment is shown in Fig. 3. Randomization, concealment and the occurrence of baseline differences were adequately described and free of risk of bias in six studies [34, 35, 37–44, 46]. Three studies were at high risk of bias due to deviations from the intervention [45, 46, 49]. Ren et al. [45] state their study is double blind, but no placebo protocol is mentioned. Two others were at high risk of bias because of troubles with adherence [46, 49], one of which performed a per protocol analysis to assure a better estimate of the treatment effect [49]. Missing data led to a high risk of bias, except in two studies [33, 41–44]. The studies all used validated outcome measurements, resulting in a low risk of bias in the domain of measurement of the outcome. In five studies, (some) outcome assessors were clearly blinded [37–39, 41–45, 49]. The selection of the reported results was often not described; only two studies published a protocol describing the analysis plan [38, 39, 41–44]. Two studies used multiple eligible analyses of the data that resulted in a high risk of bias [29–32, 34, 35]. Another risk of bias worth mentioning was a small sample size (*n* =  < 30) in three studies [33, 45, 49].

### The effect of exercise interventions

#### Physical functioning

Cardiorespiratory fitness was measured in five studies with the Six-Minute Walk Test [29–32, 37, 46–49] and aerobic capacity in a submaximal ergometer cycle test in six studies [27, 28, 38–44, 46, 49]. The 6-min walking distance improved significantly (SMD 0.41, 95% CI 0.14 to 0.68, Suppl. Figure 4A). Peak aerobic capacity did not show significant improvement in the meta-analysed studies (SMD 0.09, 95% CI − 0.30 to 0.48, Suppl. Figure 4B).

Strength was assessed in multiple studies with hand grip strength (*n* = 6) [33–35, 37–39, 41–44, 46], and various methods of upper (*n* = 3) [34, 35, 46–48] and lower (*n* = 6) extremity strength [27, 28, 37–44, 47, 48]. Hand grip strength did not improve significantly (SMD 0.15, 95% CI − 0.18 to 0.48, Suppl. Figure 5A). Overall, exercise interventions provided a small positive effect on lower extremity strength (SMD 0.35, 95% CI 0.13 to 0.56, Suppl. Figure 5B). The positive effect on upper extremity strength was not significant (SMD 0.56, 95% CI − 0.04 to 1.16, Suppl. Figure 5C).

Functional performance was measured with the 30 Second-Chair Stand test (*n* = 4) [33–35, 41–44, 46]. The effect on functional performance was not significant (MD 1.89 repetitions/second, 95% CI − 0.30 to 4.09, Suppl. Figure 6). Several other methods were used to measure physical fitness, but they were only used by one or two studies and were therefore not considered in this review.

#### Quality of Life

Eight studies assessed QOL using (parts of) the European Organization for Research and Treatment for Cancer Quality of Life Questionnaire (EORTC QLQ-C30) [27, 28, 33–35, 37, 40–44, 46–48]. The pooled result for seven trials (*n* = 437) showed a small effect on global QOL when compared to usual care (SMD 0.27, 95% CI 0.09 to 0.45, Suppl. Figure 7A). No significant effect was found for the subscale physical functioning (SMD 0.20, 95% CI − 0.10 to 0.50, Suppl. Figure 7B). The scales of emotional functioning and cognitive functioning did show a small effect (Suppl. Figure 7D and 7E). No effect was found for role functioning and social functioning (Suppl. Figure 7C and 7F).

The pooled results for the symptom scales in five trials (*n* = 247) showed a small effect for pain and diarrhoea (Supplementary Fig. 1B and 1G). Other symptom scales, namely, nausea and vomiting, dyspnoea, insomnia, appetite loss, constipation and financial difficulties (*n* = 4), did not show a significant effect (Supplementary Fig. 1A, 1C, 1D, 1E, 1F and 1H).

#### Fatigue

The effect of exercise interventions on fatigue, measured with the EORTC QLQ-C30 fatigue subscale, showed a statistically significant decrease (SMD − 0.33, 95% CI − 0.55 to − 0.11, Suppl. Figure 8A). Nine studies measured fatigue with another method than the EORTC QLQ-C30 fatigue subscale [29–35, 37–44, 46–48]. Commonly used methods were the Multidimensional Fatigue Inventory (MFI) (*n* = 4) [40–44, 46–48] and several versions of the Functional Assessment of Cancer Therapy (FACT) (*n* = 4) [29–32, 37–39, 46]. Neither showed a significant effect on fatigue (Suppl. Figure 8B and 8C).

#### Body fat and weight

Four studies reported a variety of outcome measurements accumulated by body composition measurement [37–40, 46]. The effect on body fat was not significant (SMD 0.01, 95% CI − 0.30 to 0.31, Supplementary Fig. 2A). Weight was assessed by three studies [34, 35, 37–39]. It decreased in the exercise group compared to the usual care group, but this effect was not significant (MD − 2.45 kg, 95% CI − 6.48 to1.59, Supplementary Fig. 3B).

#### Clinical outcomes

Five studies reported LOS [27, 28, 34, 35, 40, 46–48]. Two studies could not be meta-analysed due to insufficient data provision [40, 47, 48]. Meta-analysis (3 studies, *n* = 147) showed no significant difference between groups (SMD -0.17, 95% CI − 0.45 to 0.12, Suppl. Figure 9A). Mortality was similar amongst groups (RR 0.97, 95% CI 0.62 to 1.53, Suppl. Figure 9B) [27, 28, 33, 37–44, 46–49]. Three studies reported GvHD rates; rates did not significantly differ across groups (RR 1.10, 95% CI 0.78 to 1.53, Suppl. Figure 9C) [27, 28, 46–48].

### The effect of nutrition interventions

Because only two nutrition studies were included, meta-analysis was not performed. Both nutrition studies assessed hand grip strength. Ren et al. showed a significant improvement in hand grip strength (*p* =  < 0.05) [45], whereas Jabbour et al. found no between groups difference (*p* = 0.96) [36]. Furthermore, Jabbour assessed Fat Mass Index through bioimpedance analysis and LOS; but found no significant between-group effects [36].

## Discussion

This systematic review and meta-analysis includes data from thirteen studies implementing with exercise and nutrition intervention into the care of HSCT recipients aiming to improve their physical functioning. The exercise interventions were considered effective on 6-min walking distance, lower extremity strength and global QOL. No statistically significant effects were found for all other outcomes. To draw a conclusion based on the scarce results of the nutrition studies would be precarious. Overall, this meta-analysis shows that exercise interventions may be beneficial on physical functioning and QOL for HSCT patients.

These results are in line with an earlier review on the effect of exercise in HSCT recipients on physical functioning [50]. The small effect on QOL seen in our review is supported by two other meta-analyses about exercise interventions in HSCT recipients [14, 50]. Medium positive effects of exercise on fatigue found by a 2013 meta-analysis on exercise interventions in HSCT recipients were not observed in our review [50]. Comparable studies for the nutritional studies are lacking; therefore, the effect of nutrition interventions remains unclear after our review. However, combined nutrition and exercise interventions have shown potential in studies with cancer patients previously [51, 52].

Remarkably, the exercise interventions in this meta-analysis showed large heterogeneity in the method, intensity, timing and duration of the exercise. The usual care also differed. For example, Wiskemann et al. offered the control group physiotherapy [47, 48].

A major strength of this study is that it includes a specific patient population. The study population is very homogeneous, with almost solely patients with a haematological malignancy that are treated with HSCT. In addition, this study is the first systematic review to combine both exercise and nutrition interventions in one review. Compared to two previous systematic reviews, it includes five new exercise studies that were not studied in a meta-analysis before [13, 50]. During the process of writing this review, one newly published exercise intervention study was brought to the attention. The results of this RCT confirm the results of our review [53]. However, our review also has some limitations. Heterogeneity amongst the trials was present in the type of intervention and used outcome measurement methods. The latter was especially a concern in the measurement of lower and upper extremity strength. To determine the effect of a specific intervention, subgroup analysis would be necessary. The heterogeneity of interventions, methods, measurement and the small sample sizes resulted in a lack of power for subgroup analyses. Lacking information on adherence and poor adherence to the assigned intervention itself is another factor possibly clouding the results. Furthermore, the included studies were troubled with bias; most studies had a fairly large amount of missing data and drop outs. However, the majority of the patients dropped out because they had deceased, which unfortunately reflects clinical practice. Small sample sizes in the included studies might not be representative for the population. Lastly, only short-term effects were assessed.

This meta-analysis suggests that physical functioning can be improved using exercise interventions. An improved physical functioning has the potential to reduce fatigue and improve QOL [54, 55]. Lower levels of fatigue and a better QOL will provide more opportunities to furthermore sustain, or even improve, physical function throughout intensive HSCT treatment. The potential merits of nutritional interventions on physical function, QOL, fatigue level and even clinical parameters are worth investing in. The most effective intervention for improving physical functioning in HSCT recipients could be uncovered by designing trials with different types of exercise interventions, frequency, intensity, timing and duration.

Furthermore, nutrition interventions should be studied as a separate entity, as results on the effect of nutrition are lacking in this specific patient population. High-quality, heterogeneous RCTs with bigger sample sizes and longer follow-up periods, using equal measurement instruments for all relevant outcomes, are needed.

This systematic review and meta-analysis shows that exercise interventions are promising to improve short-term physical functioning in HSCT recipients. To provide optimal supportive care and decrease negative consequences of life-saving treatment, further research into the optimal exercise interventions and the potential benefit of nutrition interventions is needed.

## Supplementary Information

Below is the link to the electronic supplementary material.

Supplementary file1 (PDF 1318 KB)

Supplementary file2 (PDF 391 KB)

Supplementary file3 (PDF 361 KB)

Supplementary file4 (PDF 403 KB)

Supplementary file5 (PDF 381 KB)

Supplementary file6 (PDF 312 KB)

Supplementary file7 (PDF 336 KB)

Supplementary file8 (PDF 463 KB)

Supplementary file9 (PDF 472 KB)

Supplementary file10 (PDF 440 KB)

Supplementary file11 (PDF 445 KB)

Supplementary file12 (PDF 453 KB)

Supplementary file13 (PDF 440 KB)

Supplementary file14 (PDF 381 KB)

Supplementary file15 (PDF 336 KB)

Supplementary file16 (PDF 341 KB)

Supplementary file17 (PDF 338 KB)

Supplementary file18 (PDF 333 KB)

Supplementary file19 (PDF 292 KB)

## Data Availability

Data is available.

## References

[1] Niederwieser D, Baldomero H, Szer J, Gratwohl M, Aljurf M, Atsuta Y, Bouzas LF, Confer D, Greinix H, Horowitz M, Iida M, Lipton J, Mohty M, Novitzky N, Nunez J, Passweg J, Pasquini MC, Kodera Y, Apperley J, Seber A, Gratwohl A (2016). Hematopoietic stem cell transplantation activity worldwide in 2012 and a SWOT analysis of the Worldwide Network for Blood and Marrow Transplantation Group including the global survey. Bone Marrow Transplant.

[2] Gratwohl A, Pasquini MC, Aljurf M, Atsuta Y, Baldomero H, Foeken L, Gratwohl M, Bouzas LF, Confer D, Frauendorfer K, Gluckman E, Greinix H, Horowitz M, Iida M, Lipton J, Madrigal A, Mohty M, Noel L, Novitzky N, Nunez J, Oudshoorn M, Passweg J, van Rood J, Szer J, Blume K, Appelbaum FR, Kodera Y, Niederwieser D, Marrow T, Worldwide Network for B (2015). One million haemopoietic stem-cell transplants: a retrospective observational study. Lancet Haematol.

[3] Bhatia S (2011) Long-term health impacts of hematopoietic stem cell transplantation inform recommendations for follow-up. Expert Rev Hematol 4(4):437-452. quiz 453-434. 10.1586/ehm.11.39 10.1586/ehm.11.39 PMC3163085 21801135

[4] Chaudhry HM, Bruce AJ, Wolf RC, Litzow MR, Hogan WJ, Patnaik MS, Kremers WK, Phillips GL, Hashmi SK (2016). The incidence and severity of oral mucositis among allogeneic hematopoietic stem cell transplantation patients: a systematic review. Biol Blood Marrow Transplant.

[5] Taskinen M, Ryhänen S, Vettenranta K (2017). Graft-versus-host disease in stem cell transplantation. Duodecim.

[6] Persoon S, Kersten MJ, Buffart LM, Vander Slagmolen G, Baars JW, Visser O, Manenschijn A, Nollet F, Chinapaw MJM (2017). Health-related physical fitness in patients with multiple myeloma or lymphoma recently treated with autologous stem cell transplantation. J Sci Med Sport.

[7] Cheon J, Lee YJ, Jo JC, Kweon K, Koh S, Min YJ, Park SH, Lee SH, Kim HJ, Choi Y (2020) Late complications and quality of life assessment for survivors receiving allogeneic hematopoietic stem cell transplantation. Support Care Cancer 10.1007/s00520-020-05572-0 32556712

[8] Smeland KB, Loge JH, Aass HCD, Aspelin T, Bersvendsen H, Bolstad N, Fagerli UM, Falk RS, Fluge Ø, Fosså A, Holte H, Lund MB, Murbræch K, Reinertsen KV, Stenehjem JS, Kiserud CE (2019). Chronic fatigue is highly prevalent in survivors of autologous stem cell transplantation and associated with IL-6, neuroticism, cardiorespiratory fitness, and obesity. Bone Marrow Transplant.

[9] Mosher CE, Redd WH, Rini CM, Burkhalter JE, DuHamel KN (2009). Physical, psychological, and social sequelae following hematopoietic stem cell transplantation: a review of the literature. Psychooncology.

[10] Morishita S, Kaida K, Yamauchi S, Wakasugi T, Ikegame K, Ogawa H, Domen K (2017) Relationship of physical activity with physical function and health-related quality of life in patients having undergone allogeneic haematopoietic stem-cell transplantation. Eur J Cancer Care (Engl) 26(4). 10.1111/ecc.12669 10.1111/ecc.12669 28220548

[11] de Almeida LB, Mira PAD, Fioritto AP, Malaguti C, Neto AEH, Trevizan PF, Laterza MC, Martinez DG (2019). Functional capacity change impacts the quality of life of hospitalized patients undergoing hematopoietic stem cell transplantation. Am J Phys Med Rehabil.

[12] White LL, Kupzyk KA, Berger AM, Cohen MZ, Bierman PJ (2019). Self-efficacy for symptom management in the acute phase of hematopoietic stem cell transplant: a pilot study. Eur J Oncol Nurs.

[13] Knips L, Bergenthal N, Streckmann F, Monsef I, Elter T, Skoetz N (2019) Aerobic physical exercise for adult patients with haematological malignancies. Cochrane Database Syst Rev 1:CD009075. 10.1002/14651858.CD009075.pub3 10.1002/14651858.CD009075.pub3 PMC6354325 30702150

[14] Liang Y, Zhou M, Wang F, Wu Z (2018). Exercise for physical fitness, fatigue and quality of life of patients undergoing hematopoietic stem cell transplantation: a meta-analysis of randomized controlled trials. Jpn J Clin Oncol.

[15] Baumgartner A, Bargetzi A, Zueger N, Bargetzi M, Medinger M, Bounoure L, Gomes F, Stanga Z, Mueller B, Schuetz P (2017). Revisiting nutritional support for allogeneic hematologic stem cell transplantation-a systematic review. Bone Marrow Transplant.

[16] Murray SM, Pindoria S (2017) Nutrition support for bone marrow transplant patients. Cochrane Database Syst Rev (3). 10.1002/14651858.CD002920.pub4 10.1002/14651858.CD002920 12076459

[17] de van der Schueren MAE, Laviano A, Blanchard H, Jourdan M, Arends J, Baracos VE (2018). Systematic review and meta-analysis of the evidence for oral nutritional intervention on nutritional and clinical outcomes during chemo(radio)therapy: current evidence and guidance for design of future trials. Ann Oncol.

[18] Langius JA, Zandbergen MC, Eerenstein SE, van Tulder MW, Leemans CR, Kramer MH, Weijs PJ (2013). Effect of nutritional interventions on nutritional status, quality of life and mortality in patients with head and neck cancer receiving (chemo)radiotherapy: a systematic review. Clin Nutr.

[19] Schmitz KH, Campbell AM, Stuiver MM, Pinto BM, Schwartz AL, Morris GS, Ligibel JA, Cheville A, Galvão DA, Alfano CM, Patel AV, Hue T, Gerber LH, Sallis R, Gusani NJ, Stout NL, Chan L, Flowers F, Doyle C, Helmrich S, Bain W, Sokolof J, Winters-Stone KM, Campbell KL, Matthews CE (2019). Exercise is medicine in oncology: engaging clinicians to help patients move through cancer. CA Cancer J Clin.

[20] Vijayvergia N, Denlinger CS (2015). Lifestyle factors in cancer survivorship: where we are and where we are headed. J Pers Med.

[21] Moher D, Liberati A, Tetzlaff J, Altman DG (2009). Preferred reporting items for systematic reviews and meta-analyses: the PRISMA statement. PLoS Med.

[22] Li T, Higgins JPT, Deeks JJ (eds) (2019) Chapter 5: Collecting data. In: Higgins JPT, Thomas J, Chandler J, Cumpston M, Li T, Page MJ, Welch VA (eds). Cochrane Handbook for Systematic Reviews of Interventions, version 6.0 (updated July 2019). Cochrane. Available from www.training.cochrane.org/handbook

[23] Ouzzani M, Hammady H, Fedorowicz Z, Elmagarmid A (2016). Rayyan—a web and mobile app for systematic reviews. Systems Control Found Appl.

[24] Review Manager (RevMan) [Computer program]. Version 5.4. The Cochrane Collaboration

[25] Deeks JJ, Higgins JPT, Altman DG (eds) (2019) Chapter 10: analysing data and undertaking meta-analyses. In: Higgins JPT, Thomas J, Chandler J, Cumpston M, Li T, Page MJ, Welch VA (eds). Cochrane Handbook for Systematic Reviews of Interventions, version 6.0 (updated July 2019). Cochrane. Available from www.training.cochrane.org/handbook. In

[26] Cohen J. Statistical power analysis in the behavioral sciences. 2nd edition. Hillsdale: Lawrence Erlbaum Associates I

[27] Baumann FT, Kraut L, Schüle K, Bloch W, Fauser AA (2010). A controlled randomized study examining the effects of exercise therapy on patients undergoing haematopoietic stem cell transplantation. Bone Marrow Transplant.

[28] Baumann FT, Zopf EM, Nykamp E, Kraut L, Schüle K, Elter T, Fauser AA, Bloch W (2011). Physical activity for patients undergoing an allogeneic hematopoietic stem cell transplantation: benefits of a moderate exercise intervention. Eur J Haematol.

[29] Coleman EA, Goodwin JA, Kennedy R, Coon SK, Richards K, Enderlin C, Stewart CB, McNatt P, Lockhart K, Anaissie EJ (2012). Effects of exercise on fatigue, sleep, and performance: a randomized trial. Oncol Nurs Forum.

[30] Coleman E, Anaissie E, Coon S, Stewart C, Shaw J, Barlogie B (2004). A randomized trial of home-based exercise for patients receiving aggressive treatment and epoetin alfa for multiple myeloma: Hemoglobin (Hb), transfusion, fatigue and performance as outcomes. J Clin Oncol.

[31] Coleman EA, Coon EK, Kennedy R, Lockhart K, Anaissie EJ, Barlogie B (2006). Benefits of exercise in combination with epoetin alfa for multiple myeloma. J Clin Oncol.

[32] Coleman EA, Coon SK, Kennedy RL, Lockhart KD, Stewart CB, Anaissie EJ, Barlogie B (2008). Effects of exercise in combination with epoetin alfa during high-dose chemotherapy and autologous peripheral blood stem cell transplantation for multiple myeloma. Oncol Nurs Forum.

[33] Hacker ED, Larson J, Kujath A, Peace D, Rondelli D, Gaston L (2011). Strength training following hematopoietic stem cell transplantation. Cancer Nurs.

[34] Hacker ED, Collins E, Park C, Peters T, Patel P, Rondelli D (2017). Strength training to enhance early recovery after hematopoietic stem cell transplantation. Biol Blood Marrow Transplant.

[35] Peters T, Erdmann R, Hacker ED (2018). Exercise intervention: attrition, compliance, adherence, and progression following hematopoietic stem cell transplantation. Clin J Oncol Nurs.

[36] Jabbour J, Manana B, Sakr M, Zahreddine A, Tamim H, Bazarbachi A, Blaise D, El-Cheikh J (2019). The impact of counseling on nutritional status among hematopoietic stem cell recipients: results of a randomized controlled trial. Bone Marrow Transplant.

[37] Knols RH, de Bruin ED, Uebelhart D, Aufdemkampe G, Schanz U, Stenner-Liewen F, Hitz F, Taverna C, Aaronson NK (2011). Effects of an outpatient physical exercise program on hematopoietic stem-cell transplantation recipients: a randomized clinical trial. Bone Marrow Transplant.

[38] Koutoukidis DA, Land J, Hackshaw A, Heinrich M, McCourt O, Beeken RJ, Philpott S, DeSilva D, Rismani A, Rabin N, Popat R, Kyriakou C, Papanikolaou X, Mehta A, Paton B, Fisher A, Yong KL (2020). Fatigue, quality of life and physical fitness following an exercise intervention in multiple myeloma survivors (MASCOT): an exploratory randomised Phase 2 trial utilising a modified Zelen design. Br J Cancer.

[39] Land J, McCourt O, Heinrich M, Beeken RJ, Koutoukidis DA, Paton B, Yong K, Hackshaw A, Fisher A (2020). The adapted Zelen was a feasible design to trial exercise in myeloma survivors. J Clin Epidemiol.

[40] Pahl A, Wehrle A, Kneis S, Gollhofer A, Bertz H (2020). Whole body vibration training during allogeneic hematopoietic cell transplantation-the effects on patients' physical capacity. Ann Hematol.

[41] Persoon S, Chin AMJM, Buffart LM, Liu RDK, Wijermans P, Koene HR, Minnema MC, Lugtenburg PJ, Marijt EWA, Brug J, Nollet F, Kersten MJ (2017). Randomized controlled trial on the effects of a supervised high intensity exercise program in patients with a hematologic malignancy treated with autologous stem cell transplantation: Results from the EXIST study. PLoS ONE.

[42] Persoon S, Kersten MJ, Chinapaw MJ, Buffart LM, Burghout H, Schep G, Brug J, Nollet F (2010). Design of the EXercise Intervention after Stem cell Transplantation (EXIST) study: a randomized controlled trial to evaluate the effectiveness and cost-effectiveness of an individualized high intensity physical exercise program on fitness and fatigue in patients with multiple myeloma or (non-) Hodgkin's lymphoma treated with high dose chemotherapy and autologous stem cell transplantation. BMC Cancer.

[43] Persoon S, Chinapaw MJM, Buffart LM, Brug J, Kersten MJ, Nollet F (2018) Lessons learnt from a process evaluation of an exercise intervention in patients treated with autologous stem cell transplantation. Eur J Cancer Care (Engl) 27(1). 10.1111/ecc.12779 10.1111/ecc.12779 PMC5813165 28960542

[44] van Dongen JM, Persoon S, Jongeneel G, Bosmans JE, Kersten MJ, Brug J, Nollet F, Chinapaw MJM, Buffart LM (2019). Long-term effectiveness and cost-effectiveness of an 18-week supervised exercise program in patients treated with autologous stem cell transplantation: results from the EXIST study. J Cancer Surviv.

[45] Ren G, Zhang J, Li M, Yi S, Xie J, Zhang H, Wang J (2017). Protein blend ingestion before allogeneic stem cell transplantation improves protein-energy malnutrition in patients with leukemia. Nutr Res.

[46] Santa Mina D, Dolan LB, Lipton JH, Au D, Camacho Perez E, Franzese A, Alibhai SMH, Jones JM, Chang E (2020) Exercise before, during, and after hospitalization for allogeneic hematological stem cell transplant: a feasibility randomized controlled trial. J Clin Med 9(6). 10.3390/jcm9061854 10.3390/jcm9061854 PMC7355733 32545872

[47] Wiskemann J, Dreger P, Schwerdtfeger R, Bondong A, Huber G, Kleindienst N, Ulrich CM, Bohus M (2011). Effects of a partly self-administered exercise program before, during, and after allogeneic stem cell transplantation. Blood.

[48] Wiskemann J, Kleindienst N, Kuehl R, Dreger P, Schwerdtfeger R, Bohus M (2015). Effects of physical exercise on survival after allogeneic stem cell transplantation. Int J Cancer.

[49] Wood WA, Weaver M, Smith-Ryan AE, Hanson ED, Shea TC, Battaglini CL (2020) Lessons learned from a pilot randomized clinical trial of home-based exercise prescription before allogeneic hematopoietic cell transplantation. Support Care Cancer. 10.1007/s00520-020-05369-1 10.1007/s00520-020-05369-1 PMC7483208 32112353

[50] Persoon S, Kersten MJ, van der Weiden K, Buffart LM, Nollet F, Brug J, Chinapaw MJ (2013). Effects of exercise in patients treated with stem cell transplantation for a hematologic malignancy: a systematic review and meta-analysis. Cancer Treat Rev.

[51] Hall CC, Cook J, Maddocks M, Skipworth RJE, Fallon M, Laird BJ (2019). Combined exercise and nutritional rehabilitation in outpatients with incurable cancer: a systematic review. Support Care Cancer.

[52] Gillis C, Buhler K, Bresee L, Carli F, Gramlich L, Culos-Reed N, Sajobi TT, Fenton TR (2018). Effects of nutritional prehabilitation, with and without exercise, on outcomes of patients who undergo colorectal surgery: a systematic review and meta-analysis. Gastroenterology.

[53] Potiaumpai M, Cutrono S, Medina T, Koeppel M, Pereira D, Pirl WF, Jacobs KA, Eltoukhy M, Signorile JF (2020) Multidirectional walking in hematopoietic stem cell transplant patients. Med Sci Sports Exerc. 10.1249/mss.0000000000002474 10.1249/MSS.0000000000002474 32735114

[54] Oberoi S, Robinson PD, Cataudella D, Culos-Reed SN, Davis H, Duong N, Gibson F, Götte M, Hinds P, Nijhof SL, Tomlinson D, van der Torre P, Cabral S, Dupuis LL, Sung L (2018). Physical activity reduces fatigue in patients with cancer and hematopoietic stem cell transplant recipients: a systematic review and meta-analysis of randomized trials. Crit Rev Oncol Hematol.

[55] Mishra SI, Scherer RW, Snyder C, Geigle P, Gotay C (2015). The effectiveness of exercise interventions for improving health-related quality of life from diagnosis through active cancer treatment. Oncol Nurs Forum.

