# Supplementary material for: The effect of exercise and nutrition interventions on physical functioning in patients undergoing haematopoietic stem cell transplantation: a systematic review and meta-analysis
Source: Support Care Cancer. 2021 Jun 16;29(11):7111–26. doi: 10.1007/s00520-021-06334-2 (PMC8464580; doi:10.1007/s00520-021-06334-2)
Supplement: Supplementary file 1 — Supplementary file1 (PDF 1318 KB) [file 520_2021_6334_MOESM1_ESM.pdf]

Supplementary Material

Supplementary Appendix A. PRISMA checklist

Section/topic	#	Checklist item	Reported on page #
**TITLE**			
Title	1	Identify the report as a systematic review, meta-analysis, or both.	2
**ABSTRACT**			
Structured summary	2	Provide a structured summary including, as applicable: background; objectives; data sources; study eligibility criteria, participants, and interventions; study appraisal and synthesis methods; results; limitations; conclusions and implications of key findings; systematic review registration number.	3
**INTRODUCTION**			
Rationale	3	Describe the rationale for the review in the context of what is already known.	4
Objectives	4	Provide an explicit statement of questions being addressed with reference to participants, interventions, comparisons, outcomes, and study design (PICOS).	5
**METHODS**			
Protocol and registration	5	Indicate if a review protocol exists, if and where it can be accessed (e.g., Web address), and, if available, provide registration information including registration number.	n/a
Eligibility criteria	6	Specify study characteristics (e.g., PICOS, length of follow-up) and report characteristics (e.g., years considered, language, publication status) used as criteria for eligibility, giving rationale.	6
Information sources	7	Describe all information sources (e.g., databases with dates of coverage, contact with study authors to identify additional studies) in the search and date last searched.	6
Search	8	Present full electronic search strategy for at least one database, including any limits used, such that it could be repeated.	6
Study selection	9	State the process for selecting studies (i.e., screening, eligibility, included in systematic review, and, if applicable, included in the meta-analysis).	7
Data collection process	10	Describe method of data extraction from reports (e.g., piloted forms, independently, in duplicate) and any processes for obtaining and confirming data from investigators.	7
Data items	11	List and define all variables for which data were sought (e.g., PICOS, funding sources) and any assumptions and simplifications made.	7
Risk of bias in individual studies	12	Describe methods used for assessing risk of bias of individual studies (including specification of whether this was done at the study or outcome level), and how this information is to be used in any data synthesis.	7
Summary measures	13	State the principal summary measures (e.g., risk ratio, difference in means).	8
Synthesis of results	14	Describe the methods of handling data and combining results of studies, if done, including measures of consistency (e.g., I^2) for each meta-analysis.	8

Section/topic	#	Checklist item	Reported on page #
Risk of bias across studies	15	Specify any assessment of risk of bias that may affect the cumulative evidence (e.g., publication bias, selective reporting within studies).	n/a
Additional analyses	16	Describe methods of additional analyses (e.g., sensitivity or subgroup analyses, meta-regression), if done, indicating which were pre-specified.	n/a
**RESULTS**			
Study selection	17	Give numbers of studies screened, assessed for eligibility, and included in the review, with reasons for exclusions at each stage, ideally with a flow diagram.	9
Study characteristics	18	For each study, present characteristics for which data were extracted (e.g., study size, PICOS, follow-up period) and provide the citations.	9, T1
Risk of bias within studies	19	Present data on risk of bias of each study and, if available, any outcome level assessment (see item 12).	10
Results of individual studies	20	For all outcomes considered (benefits or harms), present, for each study: (a) simple summary data for each intervention group (b) effect estimates and confidence intervals, ideally with a forest plot.	10-12
Synthesis of results	21	Present the main results of the review. If meta-analyses are done, include for each, confidence intervals and measures of consistency	10-12
Risk of bias across studies	22	Present results of any assessment of risk of bias across studies (see Item 15).	10
Additional analysis	23	Give results of additional analyses, if done (e.g., sensitivity or subgroup analyses, meta-regression [see Item 16]).	n/a
**DISCUSSION**			
Summary of evidence	24	Summarize the main findings including the strength of evidence for each main outcome; consider their relevance to key groups (e.g., healthcare providers, users, and policy makers).	13
Limitations	25	Discuss limitations at study and outcome level (e.g., risk of bias), and at review-level (e.g., incomplete retrieval of identified research, reporting bias).	13
Conclusions	26	Provide a general interpretation of the results in the context of other evidence, and implications for future research.	14
**FUNDING**			
Funding	27	Describe sources of funding for the systematic review and other support (e.g., supply of data); role of funders for the systematic review.	n/a

From: Moher D, Liberati A, Tetzlaff J, Altman DG, The PRISMA Group (2009). Preferred Reporting Items for Systematic Reviews and Meta-Analyses: The PRISMA Statement. PLoS Med 6(7): e1000097. doi:10.1371/journal.pmed1000097

Supplementary Appendix B. Search strategy

Search strategy

PubMed

#	Searches
1	"Stem Cell Transplantation"[Mesh] OR "Bone Marrow Transplantation"[Mesh]
2	Stem cell transplant*[tiab] OR stem cell therap*[tiab] OR hematopoietic cell transplant*[tiab] OR hematopoietic cell transplant*[tiab] OR haematopoietic cell transplant*[tiab] OR haematopoietic cell therap*[tiab] OR SCT[tiab] OR HSCT[tiab] OR bone marrow transplant*[tiab] OR bone marrow therap*[tiab] OR BMT[tiab]
3	1 OR 2 [SCT terms]
4	"Exercise Movement Techniques"[Mesh:NoExp] OR "Exercise Therapy"[Mesh] OR "Exercise"[Mesh] OR "Sports"[Mesh] OR "Physical Therapy Modalities"[Mesh:NoExp]
5	Aerobic training*[tiab] OR Cardio training*[tiab] OR Cardiovascular training*[tiab] OR Endurance training*[tiab] OR Exercis*[tiab] OR Physical condition*[tiab] OR Physical intervention*[tiab] OR Physical therap*[tiab] OR Physical training*[tiab] OR Physiotherap*[tiab] OR Resistance training*[tiab] OR Strength training*[tiab] OR Training program*[tiab] OR Fitness training*[tiab] OR Muscle training*[tiab] OR Weight-Bearing[tiab] OR Weight-lifting[tiab]
6	"Nutrition Therapy"[Mesh] OR "Diet"[Mesh] OR “Nutritional Assessment”[Mesh] OR “Nutritional Support”[Mesh]
7	Artificial feeding*[tiab] OR Diet[tiab] OR Dietary[tiab] OR Dieting[tiab] OR Diets[tiab] OR Enteral feeding*[tiab] OR Intravenous Feeding*[tiab] OR Nutrition[tiab] OR Nutritional support*[tiab] OR Parenteral feeding*[tiab] OR Tube Feeding*[tiab]
8	4 OR 5 OR 6 OR 7 [intervention terms]
9	(randomized controlled trial[pt] OR controlled clinical trial[pt] OR randomized[tiab] OR placebo[tiab] OR drug therapy[sh] OR randomly[tiab] OR trial[tiab] OR groups[tiab] NOT (animals [mh] NOT humans [mh])) [RCT filter]
10	3 AND 8 AND 9 [SCT + intervention + RCT filter]

Note: the RCT filter used here is the Cochrane Highly Sensitive Search Strategy for identifying randomized trials in MEDLINE: sensitivity-maximizing version (2008 revision); PubMed format [22]

Embase

#	Searches
1	exp stem cell transplantation/ OR exp bone marrow transplantation/
2	stem cell transplant*.ti,ab,kw. OR stem cell therap*.ti,ab,kw. OR hematopoietic cell transplant*.ti,ab,kw. OR hematopoietic cell therap*.ti,ab,kw. OR haematopoietic cell transplant*.ti,ab,kw. OR haematopoietic cell therap*.ti,ab,kw. OR SCT.ti,ab,kw. OR HSCT.ti,ab,kw. OR bone marrow transplant*.ti,ab,kw. OR bone marrow therap*.ti,ab,kw. OR BMT.ti,ab,kw.
3	1 OR 2 [SCT terms]
4	exp exercise/ OR exp sport/ OR exp kinesiotherapy/ OR exp physiotherapy/
5	aerobic training*.ti,ab,kw. OR Cardio training*.ti,ab,kw. OR Cardiovascular training*.ti,ab,kw. OR Endurance training*.ti,ab,kw. OR Exercis*.ti,ab,kw. OR Physical condition*.ti,ab,kw. OR Physical intervention*.ti,ab,kw. OR Physical therap*.ti,ab,kw. OR Physical training*.ti,ab,kw. OR Physio therap*.ti,ab,kw. OR Resistance training*.ti,ab,kw. OR Strength training*.ti,ab,kw. OR Training program*.ti,ab,kw. OR Fitness training*.ti,ab,kw. OR Muscle training*.ti,ab,kw. OR Weight-Bearing.ti,ab,kw. OR Weight-lifting.ti,ab,kw.
6	exp diet therapy/ OR exp diet/ OR exp dietary intake/ OR exp nutritional assessment/ OR exp nutritional counseling/ OR exp artificial feeding/
7	artificial feeding*.ti,ab,kw. OR Diet.ti,ab,kw. OR Dietary.ti,ab,kw. OR Dieting.ti,ab,kw. OR Diets.ti,ab,kw. OR Enteral feeding*.ti,ab,kw. OR Intravenous Feeding*.ti,ab,kw. OR Nutrition.ti,ab,kw. OR Nutritional support*.ti,ab,kw. OR Parenteral feeding*.ti,ab,kw. OR Tube Feeding*.ti,ab,kw.
8	4 OR 5 OR 6 OR 7 [intervention terms]
9	Randomized controlled trial/ or Controlled clinical study/ or ((random$.ti,ab. or randomization/ or intermethod comparison/ or placebo.ti,ab. or (compare or compared or comparison).ti. or ((evaluated or evaluate or evaluating or assessed or assess) and (compare or compared or comparing or comparison)).ab. or (open adj label).ti,ab. or ((double or single or doubly or singly) adj (blind or blinded or blindly)).ti,ab. or double blind procedure/ or parallel group$1.ti,ab. or (crossover or cross over).ti,ab. or ((assign$ or match or matched or allocation) adj5 (alternate or group$1 or intervention$1 or patient$1 or subject$1 or participant$1)).ti,ab. or (assigned or allocated).ti,ab. or (controlled adj7 (study or design or trial)).ti,ab. or (volunteer or volunteers).ti,ab. or human experiment/ or trial.ti.) not (Randomized controlled trial/ or Controlled clinical study/) not (((random$ adj sampl$ adj7 ("cross section$" or questionnaire$1 or survey$ or database$1)).ti,ab. not (comparative study/ or controlled study/ or randomi?ed controlled.ti,ab. or randomly assigned.ti,ab.)) or (Cross-sectional study/ not (randomized controlled trial/ or controlled clinical study/ or controlled study/ or randomi?ed controlled.ti,ab. or control group$1.ti,ab.)) or (((case adj control$) and random$) not randomi?ed controlled).ti,ab. or (Systematic review not (trial or study)).ti. or (nonrandom$ not random$).ti,ab. or "Random field$".ti,ab. or (random cluster adj3 sampl$).ti,ab. or ((review.ab. and review.pt.) not trial.ti.) or ("we searched".ab. and (review.ti. or review.pt.)) or "update review".ab. or (databases adj4 searched).ab. or ((rat or rats or mouse or mice or swine or porcine or murine or sheep or lambs or pigs or piglets or rabbit or rabbits or cat or cats or dog or dogs or cattle or bovine or monkey or monkeys or trout or marmoset$1).ti. and animal experiment/) or (Animal experiment/ not (human experiment/ or human/)))) [RCT filter]
10	3 AND 8 AND 9 [SCT + intervention + RCT filter]
11	limit 10 to conference abstracts
12	10 NOT 11 [conference abstracts excluded]

Note: the RCT filter used here is the Cochrane Highly Sensitive Search Strategy for identifying controlled trials in Embase: (2018 revision); Ovid format.[22]

Cochrane

#	Search
1	“Stem cell transplant*”:ti,ab,kw OR “stem cell therap*:ti,ab,kw OR “hematopoietic cell transplant*”:ti,ab,kw OR “hematopoetic cell therap*”:ti,ab,kw OR “haematopoietic cell transplant*”:ti,ab,kw OR “hematopoietic cell therap*:ti,ab,kw OR SCT:ti,ab,kw OR HSCT:ti,ab,kw OR “bone marrow transplant*”:ti,ab,kw OR “bone marrow therap*”:ti,ab,kw OR BMT:ti,ab,kw
2	“Aerobic training*“:ti,ab,kw OR “Cardio training*”:ti,ab,kw OR “Cardiovascular training*”:ti,ab,kw OR “Endurance training*”:ti,ab,kw OR Exercis*:ti,ab,kw OR “Physical condition*”:ti,ab,kw OR “Physical intervention*”:ti,ab,kw OR “Physical therap*”:ti,ab,kw OR “Physical training*”:ti,ab,kw OR Physiotherap*:ti,ab,kw OR “Resistance training*”:ti,ab,kw OR “Strength training*”:ti,ab,kw OR “Training program*”:ti,ab,kw OR “Fitness training”:ti,ab,kw OR “Muscle training”:ti,ab,kw OR “Weight-Bearing”:ti,ab,kw OR “Weight-lifting”:ti,ab,kw
3	“Artificial feeding*”:ti,ab,kw OR Diet:ti,ab,kw OR Dietary:ti,ab,kw OR Dieting:ti,ab,kw OR Diets:ti,ab,kw OR “Enteral feeding*”:ti,ab,kw OR “Intravenous Feeding*”:ti,ab,kw OR Nutrition:ti,ab,kw OR “Nutritional support*”:ti,ab,kw OR “Parenteral feeding*”:ti,ab,kw OR “Tube Feeding*”:ti,ab,kw
4	2 OR 3 [intervention terms]
5	1 AND 4 [SCT + intervention]

Supplementary Figures

A. Nausea and vomiting

Study or Subgroup	Exercise Intervention Mean	SD	Total	Usual care Mean	SD	Total	Weight	Std. Mean Difference IV, Random, 95% CI
Baumann 2010	26.4	33.5	24	30.6	29.5	25	16.7%	-0.13 [-0.69, 0.43]
Hacker 2011	0	0	8	1.9	5.6	9		Not estimable
Hacker 2017	17.7	29.7	33	10.3	19.3	34	22.1%	0.29 [-0.19, 0.77]
Knols 2011	3.7	9.9	57	6.5	13.4	57	35.2%	-0.24 [-0.60, 0.13]
Wiskemann 2011	13.8	22.3	40	19.2	26.6	40	26.0%	-0.22 [-0.66, 0.22]
**Total (95% CI)**			**162**			**165**	**100.0%**	**-0.10 [-0.34, 0.14]**

Heterogeneity: $Tau^2 = 0.01$; $Chi^2 = 3.37$, $df = 3$ ($P = 0.34$); $I^2 = 11\%$
Test for overall effect: $Z = 0.80$ ($P = 0.42$)

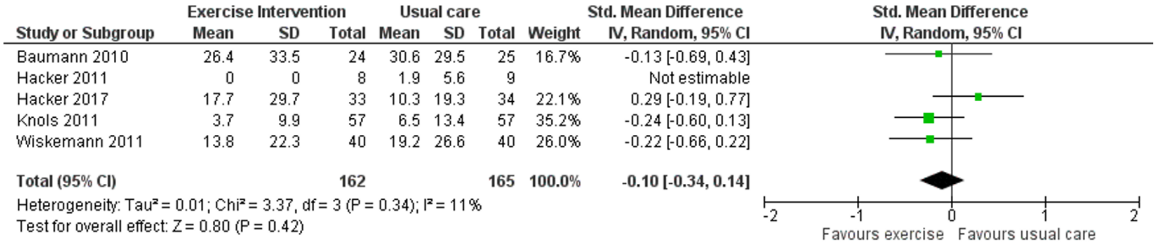

B. Pain

Study or Subgroup	Exercise Intervention Mean	SD	Total	Usual care Mean	SD	Total	Weight	Std. Mean Difference IV, Random, 95% CI
Baumann 2010	33.3	29.2	24	40.3	28.8	25	15.1%	-0.24 [-0.80, 0.32]
Hacker 2011	18.8	20.8	8	35.2	36.8	9	5.0%	-0.51 [-1.48, 0.46]
Hacker 2017	25.3	31.8	33	34.8	33.7	34	20.6%	-0.29 [-0.77, 0.20]
Knols 2011	17.8	27.1	57	26.8	28.4	57	34.9%	-0.32 [-0.69, 0.05]
Wiskemann 2011	25	31.8	40	35.8	28.9	40	24.4%	-0.35 [-0.79, 0.09]
**Total (95% CI)**			**162**			**165**	**100.0%**	**-0.32 [-0.54, -0.10]**

Heterogeneity: $Tau^2 = 0.00$; $Chi^2 = 0.27$, $df = 4$ ($P = 0.99$); $I^2 = 0\%$
Test for overall effect: $Z = 2.86$ ($P = 0.004$)

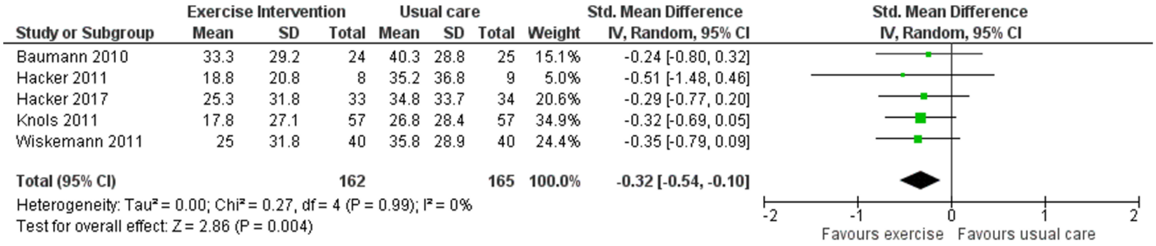

C. Dyspnoea

Study or Subgroup	Exercise Intervention Mean	SD	Total	Usual care Mean	SD	Total	Weight	Std. Mean Difference IV, Random, 95% CI
Baumann 2010	29.9	28.7	24	39.8	33.8	25	14.9%	-0.31 [-0.87, 0.25]
Hacker 2011	12.5	17.3	8	11.1	16.7	9	5.2%	0.08 [-0.87, 1.03]
Hacker 2017	20.2	28.8	33	21.6	25.8	34	20.6%	-0.05 [-0.53, 0.43]
Knols 2011	21.8	24.6	57	29.2	23	57	34.7%	-0.31 [-0.68, 0.06]
Wiskemann 2011	30	25.9	40	33.3	31.1	40	24.6%	-0.11 [-0.55, 0.32]
**Total (95% CI)**			**162**			**165**	**100.0%**	**-0.19 [-0.41, 0.03]**

Heterogeneity: $Tau^2 = 0.00$; $Chi^2 = 1.31$, $df = 4$ ($P = 0.86$); $I^2 = 0\%$
Test for overall effect: $Z = 1.69$ ($P = 0.09$)

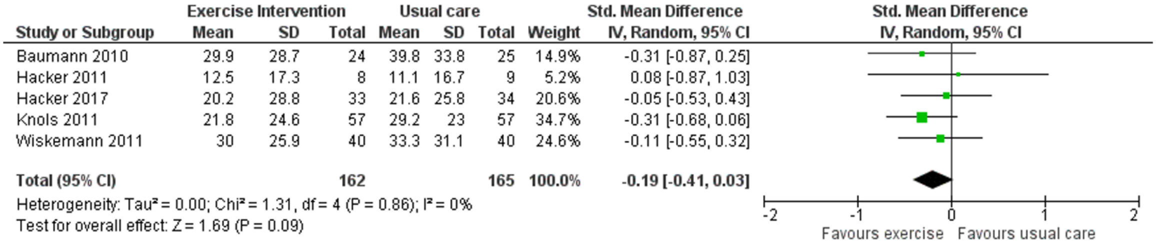

D. Vomiting

Study or Subgroup	Exercise Intervention Mean	SD	Total	Usual care Mean	SD	Total	Weight	Std. Mean Difference IV, Random, 95% CI
Baumann 2010	46	36.1	24	39.8	31.5	25	15.0%	0.18 [-0.38, 0.74]
Hacker 2011	25	23.6	8	18.5	17.6	9	5.1%	0.30 [-0.66, 1.26]
Hacker 2017	37.4	38.9	33	39.2	37.1	34	20.6%	-0.05 [-0.53, 0.43]
Knols 2011	23	27.4	57	31.5	30.8	57	34.7%	-0.29 [-0.66, 0.08]
Wiskemann 2011	30.8	32.4	40	35.8	31.5	40	24.5%	-0.15 [-0.59, 0.28]
**Total (95% CI)**			**162**			**165**	**100.0%**	**-0.11 [-0.32, 0.11]**

Heterogeneity: $Tau^2 = 0.00$; $Chi^2 = 2.74$, $df = 4$ ($P = 0.60$); $I^2 = 0\%$
Test for overall effect: $Z = 0.95$ ($P = 0.34$)

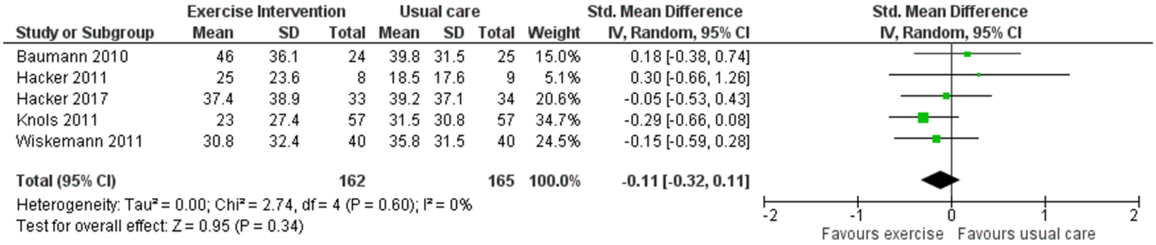

Supplementary Figure 1A, 1B, 1C and 1D. Meta-analysis for the effect of exercise interventions on symptom scales.

E. Appetite loss

Study or Subgroup	Exercise Intervention Mean	SD	Total	Usual care Mean	SD	Total	Weight	Std. Mean Difference IV, Random, 95% CI
Baumann 2010	48.3	35.2	24	45.2	33.9	25	15.0%	0.09 [-0.47, 0.65]
Hacker 2011	12.5	17.3	8	18.5	24.2	9	5.1%	-0.27 [-1.23, 0.69]
Hacker 2017	22.2	33	33	28.4	35.9	34	20.5%	-0.18 [-0.66, 0.30]
Knols 2011	8.6	17.2	57	10.7	22.1	57	35.0%	-0.11 [-0.47, 0.26]
Wiskemann 2011	31.7	35.2	40	42.5	37.7	40	24.3%	-0.29 [-0.73, 0.15]
**Total (95% CI)**			**162**			**165**	**100.0%**	**-0.15 [-0.36, 0.07]**

Heterogeneity: Tau² = 0.00; Chi² = 1.23, df = 4 (P = 0.87); I² = 0%
Test for overall effect: Z = 1.31 (P = 0.19)

Std. Mean Difference IV, Random, 95% CI

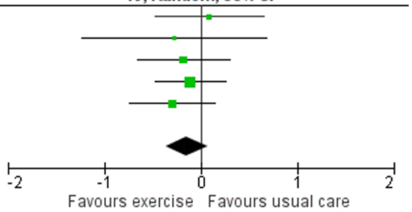

F. Constipation

Study or Subgroup	Exercise Intervention Mean	SD	Total	Usual care Mean	SD	Total	Weight	Std. Mean Difference IV, Random, 95% CI
Baumann 2010	5.9	13	24	7.5	20.6	25	15.8%	-0.09 [-0.65, 0.47]
Hacker 2011	4.2	11.8	8	0	0	9		Not estimable
Hacker 2017	8.1	22.1	33	9.8	19.3	34	21.6%	-0.08 [-0.56, 0.40]
Knols 2011	4.6	17	57	7.7	16.8	57	36.7%	-0.18 [-0.55, 0.19]
Wiskemann 2011	4.2	17.2	40	5.8	14.9	40	25.8%	-0.10 [-0.54, 0.34]
**Total (95% CI)**			**162**			**165**	**100.0%**	**-0.12 [-0.35, 0.10]**

Heterogeneity: Tau² = 0.00; Chi² = 0.15, df = 3 (P = 0.98); I² = 0%
Test for overall effect: Z = 1.09 (P = 0.27)

Std. Mean Difference IV, Random, 95% CI

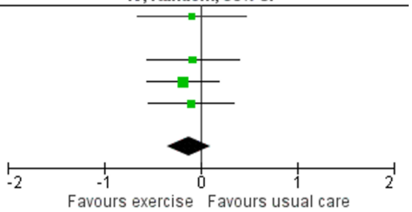

G. Diarrhoea

Study or Subgroup	Exercise Intervention Mean	SD	Total	Usual care Mean	SD	Total	Weight	Std. Mean Difference IV, Random, 95% CI
Baumann 2010	34.5	39.3	24	41.9	36.5	25	15.9%	-0.19 [-0.75, 0.37]
Hacker 2011	0	0	8	11.1	23.6	9		Not estimable
Hacker 2017	10.1	17.6	33	11.8	27.1	34	21.9%	-0.07 [-0.55, 0.41]
Knols 2011	8.6	18.3	57	18.5	24.6	57	36.3%	-0.45 [-0.83, -0.08]
Wiskemann 2011	10.8	25.5	40	18.3	27.2	40	25.9%	-0.28 [-0.72, 0.16]
**Total (95% CI)**			**162**			**165**	**100.0%**	**-0.28 [-0.51, -0.06]**

Heterogeneity: Tau² = 0.00; Chi² = 1.64, df = 3 (P = 0.65); I² = 0%
Test for overall effect: Z = 2.49 (P = 0.01)

Std. Mean Difference IV, Random, 95% CI

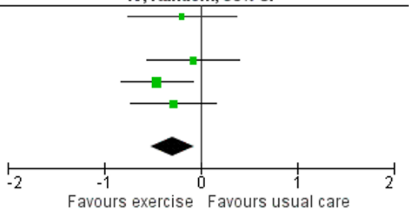

H. Financial difficulties

Study or Subgroup	Exercise Intervention Mean	SD	Total	Usual care Mean	SD	Total	Weight	Std. Mean Difference IV, Random, 95% CI
Baumann 2010	23.8	32.5	24	40.9	35.2	25	21.8%	-0.50 [-1.07, 0.07]
Hacker 2011	50	35.6	8	29.6	30.9	9	8.4%	0.58 [-0.39, 1.56]
Hacker 2017	28.3	36.4	33	29.4	36.5	34	28.5%	-0.03 [-0.51, 0.45]
Knols 2011	14.4	27.3	57	18.5	27.6	57	41.2%	-0.15 [-0.52, 0.22]
**Total (95% CI)**			**122**			**125**	**100.0%**	**-0.13 [-0.42, 0.17]**

Heterogeneity: Tau² = 0.02; Chi² = 3.81, df = 3 (P = 0.28); I² = 21%
Test for overall effect: Z = 0.85 (P = 0.39)

Std. Mean Difference IV, Random, 95% CI

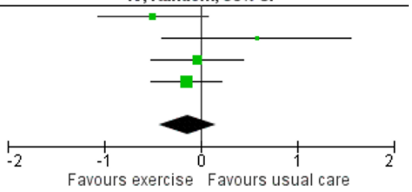

Supplementary Figure 1E, 1F, 1G and 1H. Meta-analysis for the effect of exercise interventions on symptom scales.

A. Body fat

Study or Subgroup	Exercise intervention Mean	SD	Total	Usual care Mean	SD	Total	Weight	Std. Mean Difference IV, Random, 95% CI
Knols 2011	18.3	7	57	19.6	7.4	57	42.7%	-0.18 [-0.55, 0.19]
Koutoukidis 2020	28.6	8	34	29.7	10.8	34	29.9%	-0.11 [-0.59, 0.36]
Pahl 2020	36.7	8.8	21	32	10.2	23	20.7%	0.48 [-0.12, 1.08]
Santa Mina 2020	21.7	2.7	6	21	2.6	6	6.6%	0.24 [-0.89, 1.38]
**Total (95% CI)**			**118**			**120**	**100.0%**	**0.01 [-0.30, 0.31]**

Heterogeneity: $Tau^2 = 0.02$; $Chi^2 = 3.77$, df = 3 (P = 0.29); $I^2 = 20\%$
Test for overall effect: Z = 0.03 (P = 0.97)

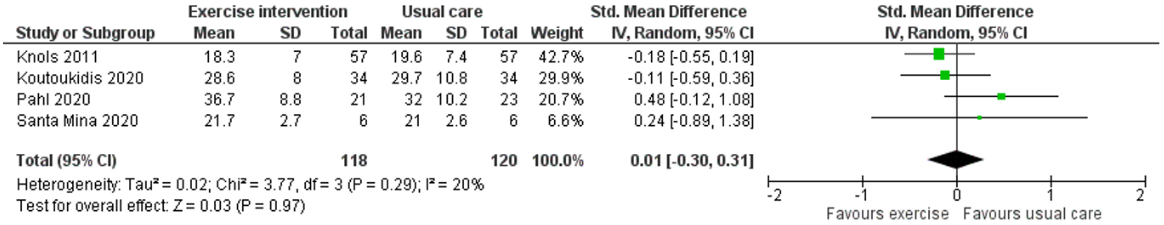

B. Weight

Study or Subgroup	Exercise intervention Mean	SD	Total	Usual care Mean	SD	Total	Weight	Mean Difference IV, Random, 95% CI
Hacker 2017	79.12	22.7	33	79.56	24.1	34	13.0%	-0.44 [-11.65, 10.77]
Knols 2011	70.8	14.5	57	72.5	13.8	57	60.2%	-1.70 [-6.90, 3.50]
Koutoukidis 2020	76.4	14.3	35	81.5	18.4	34	26.8%	-5.10 [-12.89, 2.69]
**Total (95% CI)**			**125**			**125**	**100.0%**	**-2.45 [-6.48, 1.59]**

Heterogeneity: $Tau^2 = 0.00$; $Chi^2 = 0.65$, df = 2 (P = 0.72); $I^2 = 0\%$
Test for overall effect: Z = 1.19 (P = 0.23)

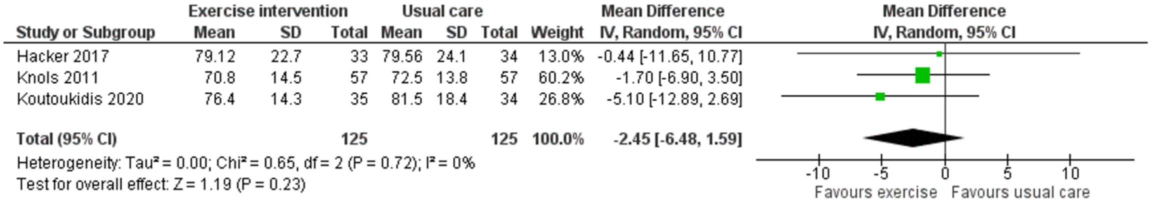

Supplementary Figure 2. Meta-analysis for the effect of exercise interventions on (A) body fat and (B) weight.
