# Supplementary figures and images for: The effect of exercise and nutrition interventions on physical functioning in patients undergoing haematopoietic stem cell transplantation: a systematic review and meta-analysis
Source: Support Care Cancer. 2021 Jun 16;29(11):7111–26. doi: 10.1007/s00520-021-06334-2 (PMC8464580; doi:10.1007/s00520-021-06334-2)

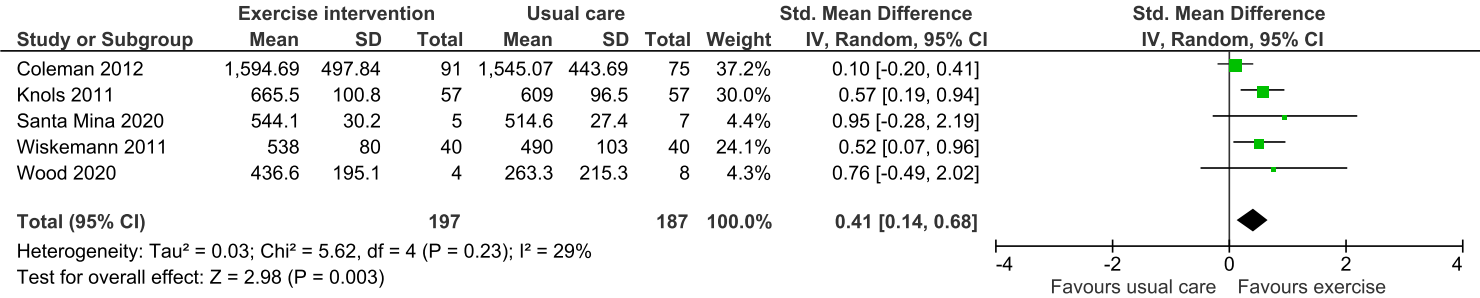

Supplement: Supplementary file 2 — Supplementary file2 (PDF 391 KB) [file 520_2021_6334_MOESM2_ESM.pdf]

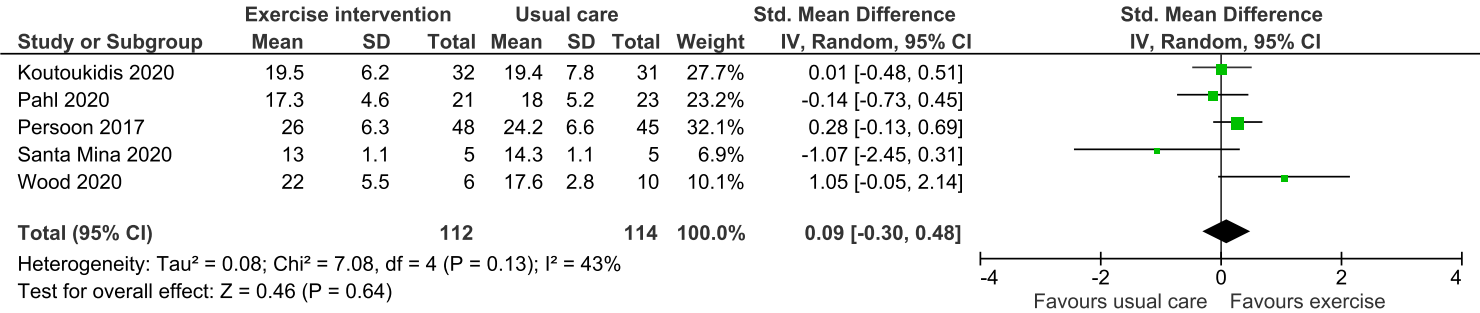

Supplement: Supplementary file 3 — Supplementary file3 (PDF 361 KB) [file 520_2021_6334_MOESM3_ESM.pdf]

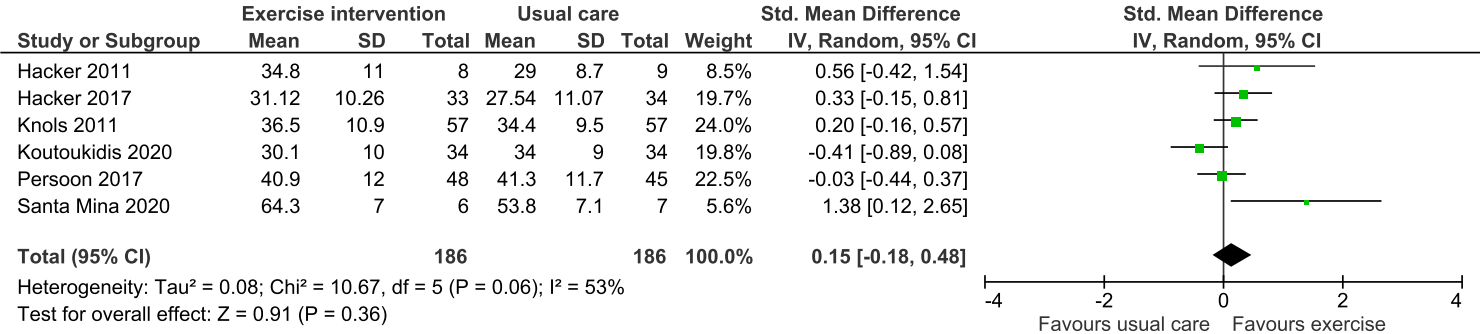

Supplement: Supplementary file 4 — Supplementary file4 (PDF 403 KB) [file 520_2021_6334_MOESM4_ESM.pdf]

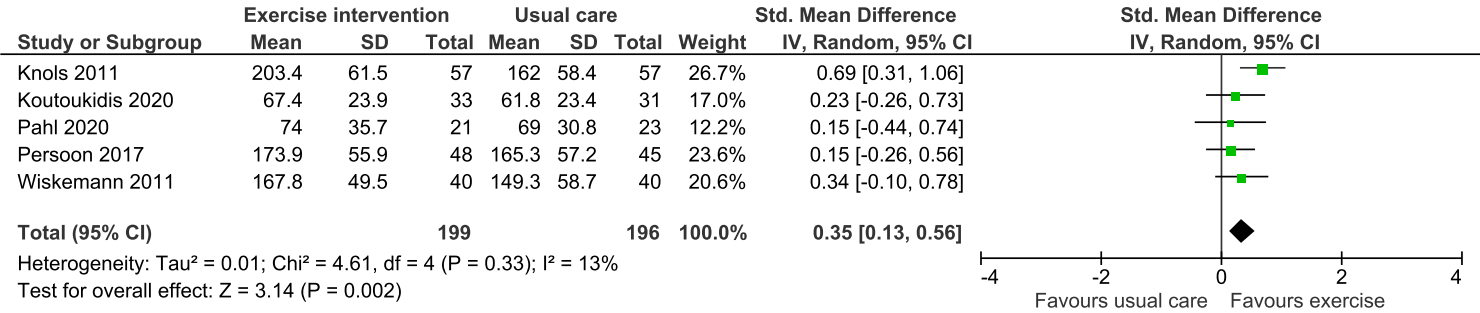

Supplement: Supplementary file 5 — Supplementary file5 (PDF 381 KB) [file 520_2021_6334_MOESM5_ESM.pdf]

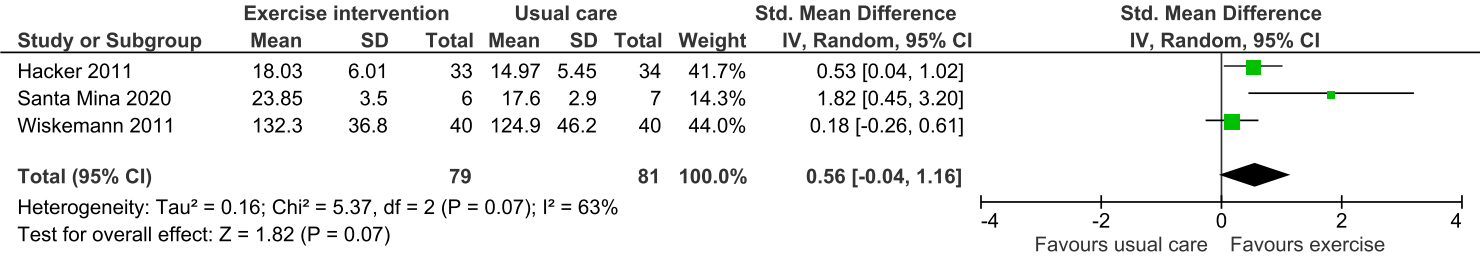

Supplement: Supplementary file 6 — Supplementary file6 (PDF 312 KB) [file 520_2021_6334_MOESM6_ESM.pdf]

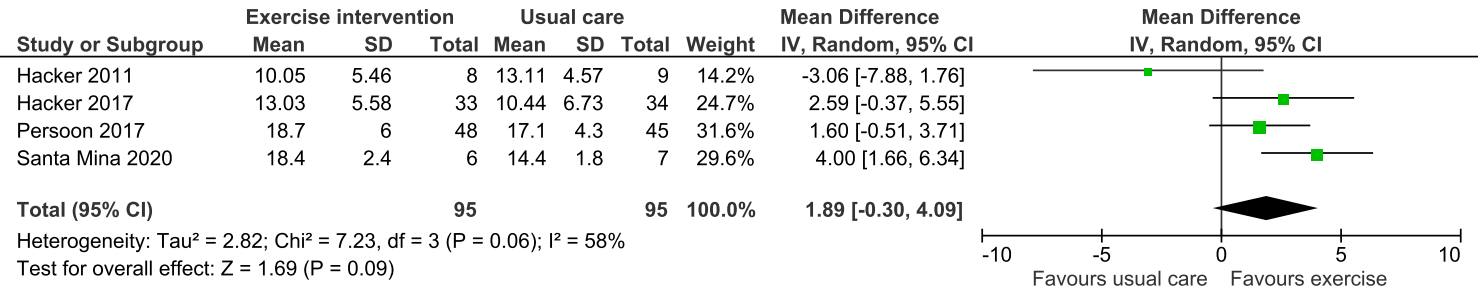

Supplement: Supplementary file 7 — Supplementary file7 (PDF 336 KB) [file 520_2021_6334_MOESM7_ESM.pdf]

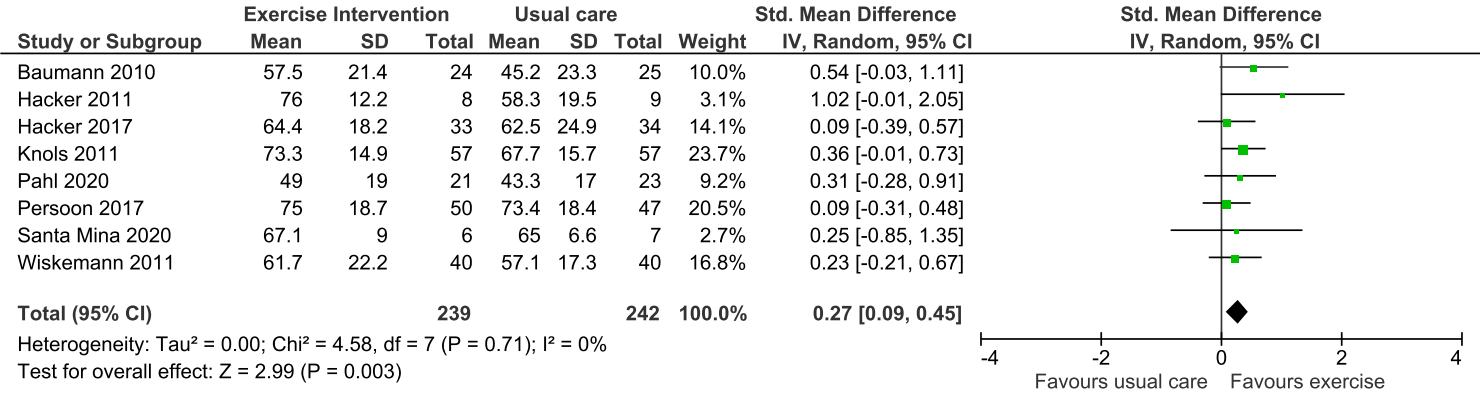

Supplement: Supplementary file 8 — Supplementary file8 (PDF 463 KB) [file 520_2021_6334_MOESM8_ESM.pdf]

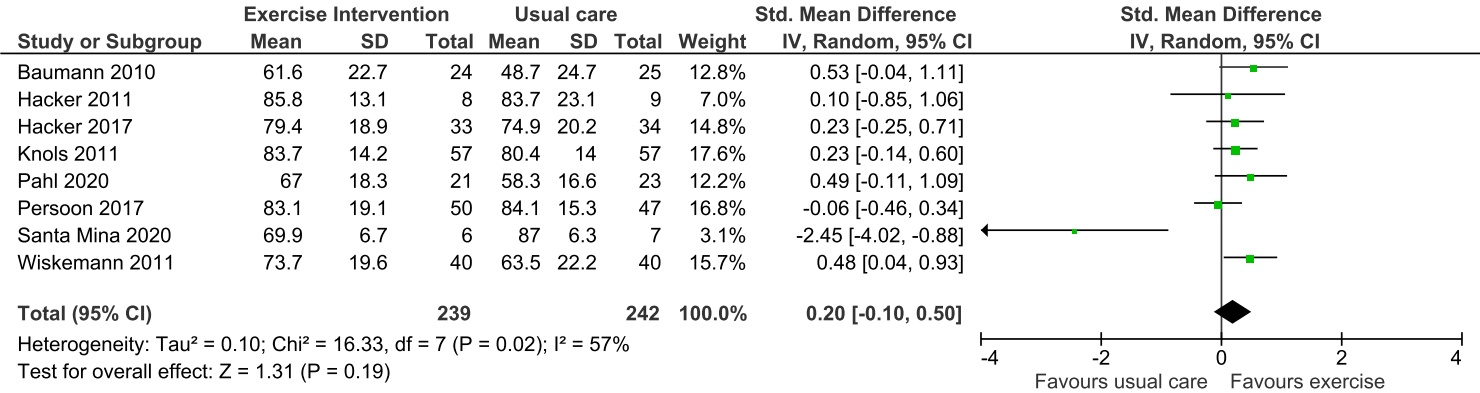

Supplement: Supplementary file 9 — Supplementary file9 (PDF 472 KB) [file 520_2021_6334_MOESM9_ESM.pdf]

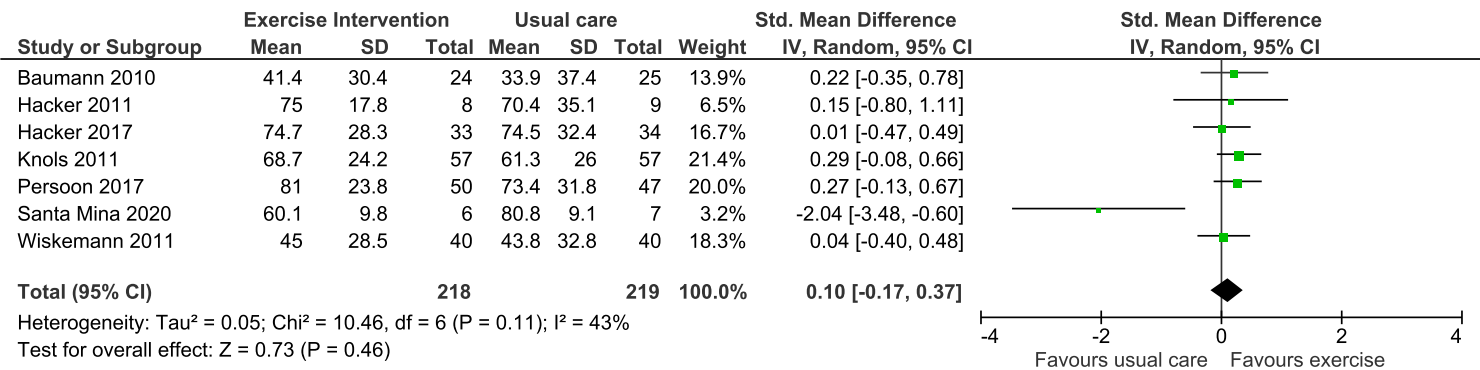

Supplement: Supplementary file 10 — Supplementary file10 (PDF 440 KB) [file 520_2021_6334_MOESM10_ESM.pdf]

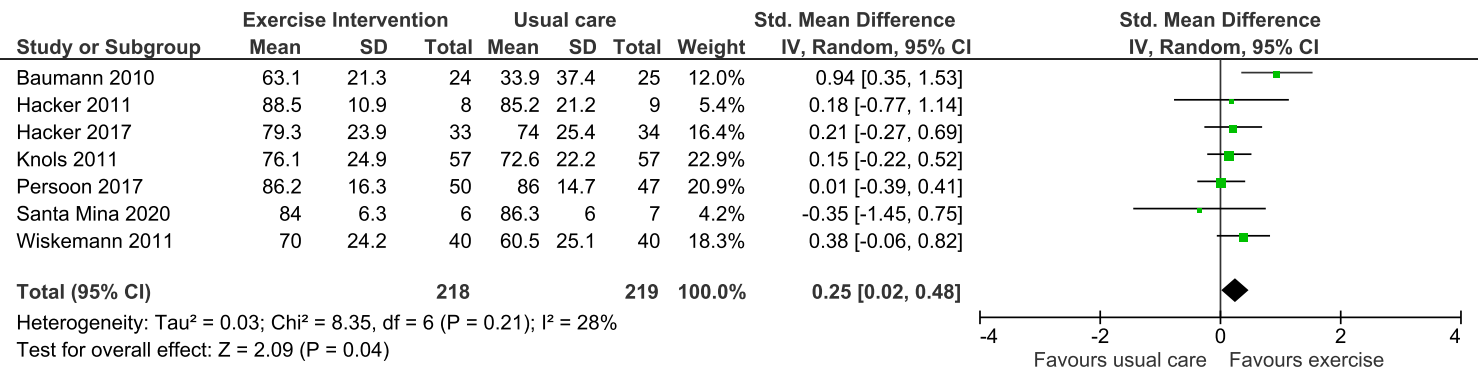

Supplement: Supplementary file 11 — Supplementary file11 (PDF 445 KB) [file 520_2021_6334_MOESM11_ESM.pdf]

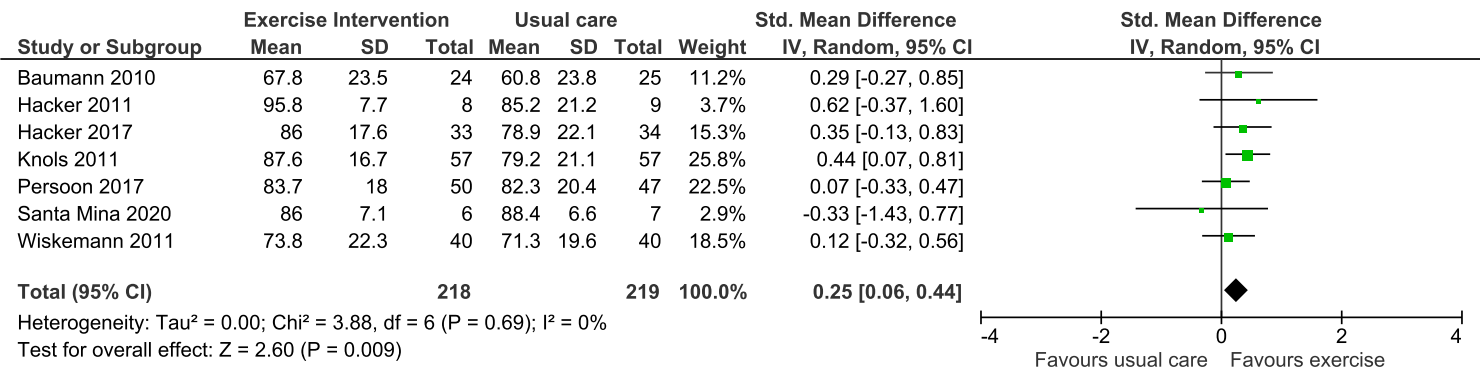

Supplement: Supplementary file 12 — Supplementary file12 (PDF 453 KB) [file 520_2021_6334_MOESM12_ESM.pdf]

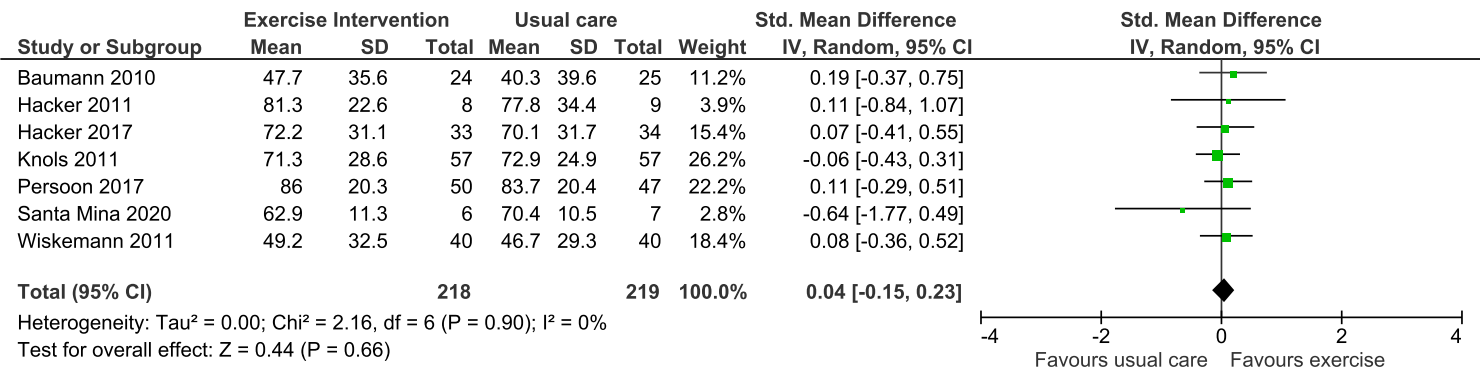

Supplement: Supplementary file 13 — Supplementary file13 (PDF 440 KB) [file 520_2021_6334_MOESM13_ESM.pdf]

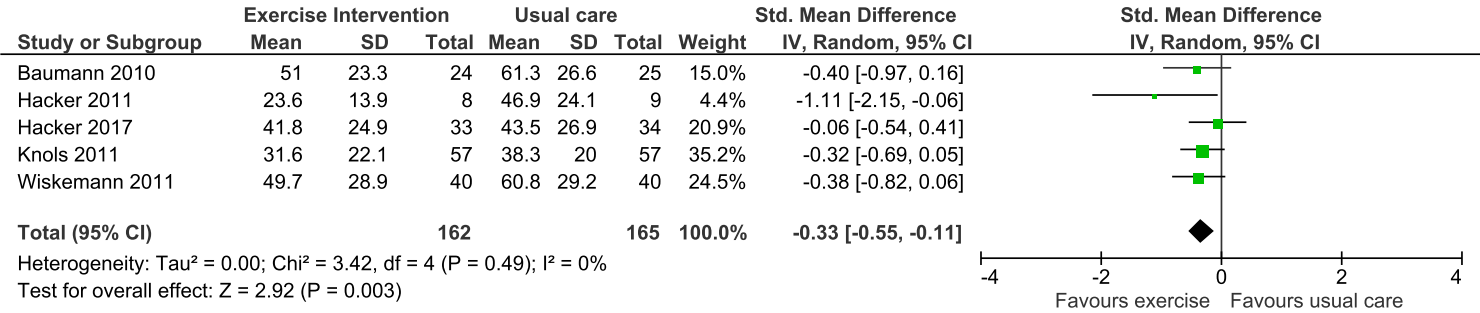

Supplement: Supplementary file 14 — Supplementary file14 (PDF 381 KB) [file 520_2021_6334_MOESM14_ESM.pdf]

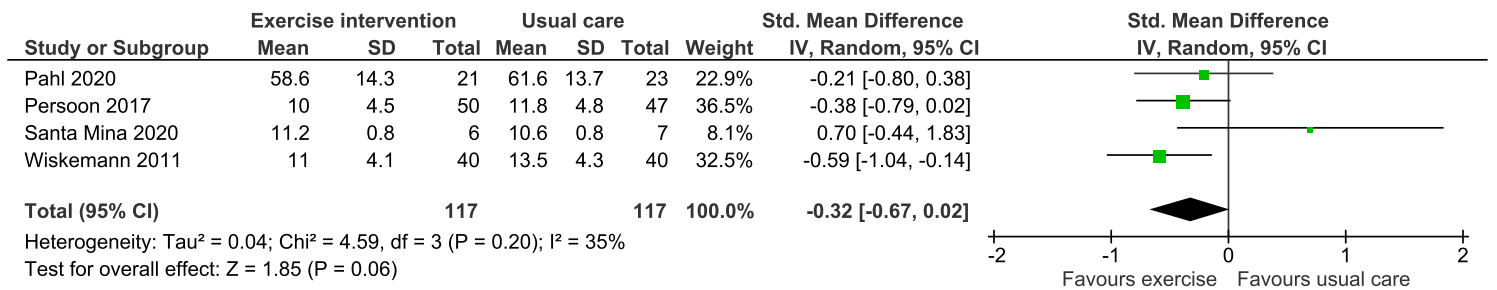

Supplement: Supplementary file 15 — Supplementary file15 (PDF 336 KB) [file 520_2021_6334_MOESM15_ESM.pdf]

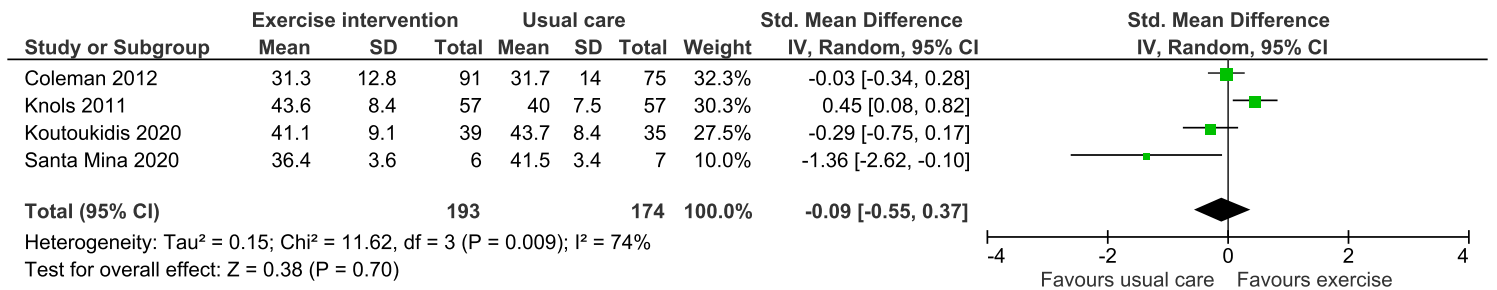

Supplement: Supplementary file 16 — Supplementary file16 (PDF 341 KB) [file 520_2021_6334_MOESM16_ESM.pdf]

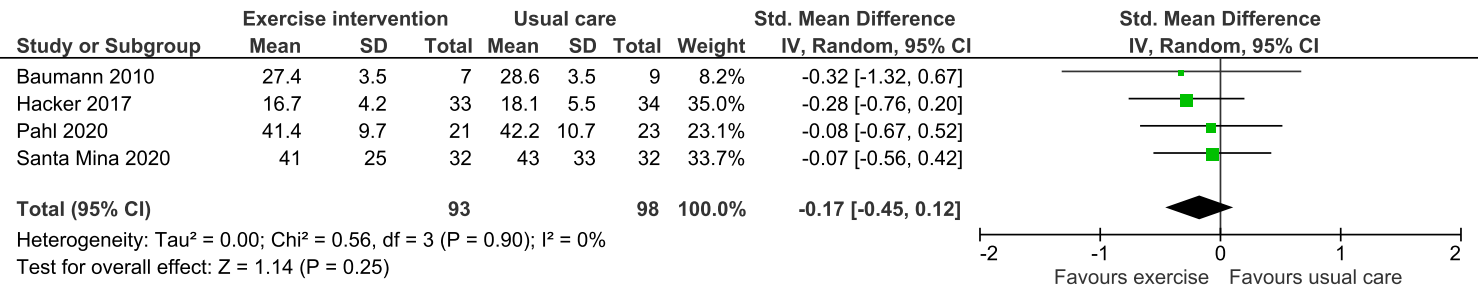

Supplement: Supplementary file 17 — Supplementary file17 (PDF 338 KB) [file 520_2021_6334_MOESM17_ESM.pdf]

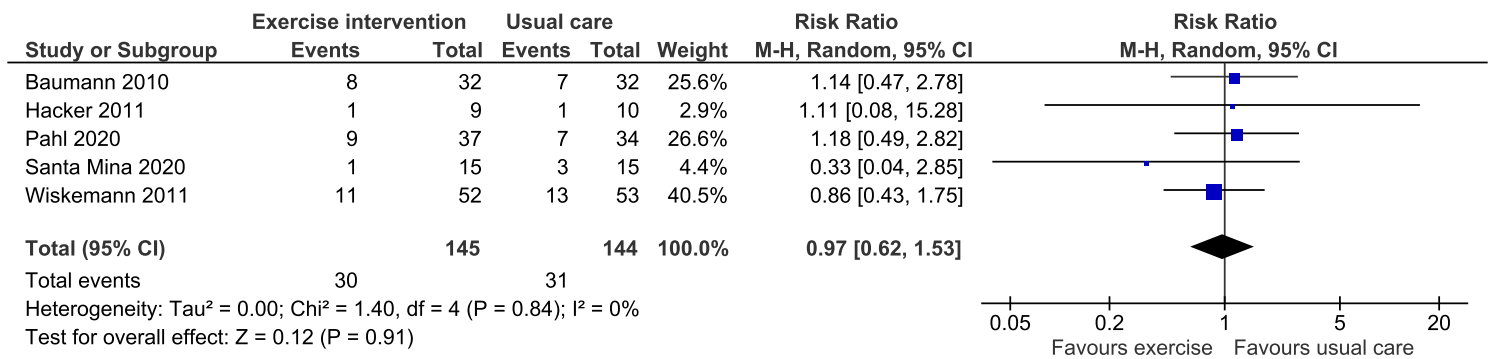

Supplement: Supplementary file 18 — Supplementary file18 (PDF 333 KB) [file 520_2021_6334_MOESM18_ESM.pdf]

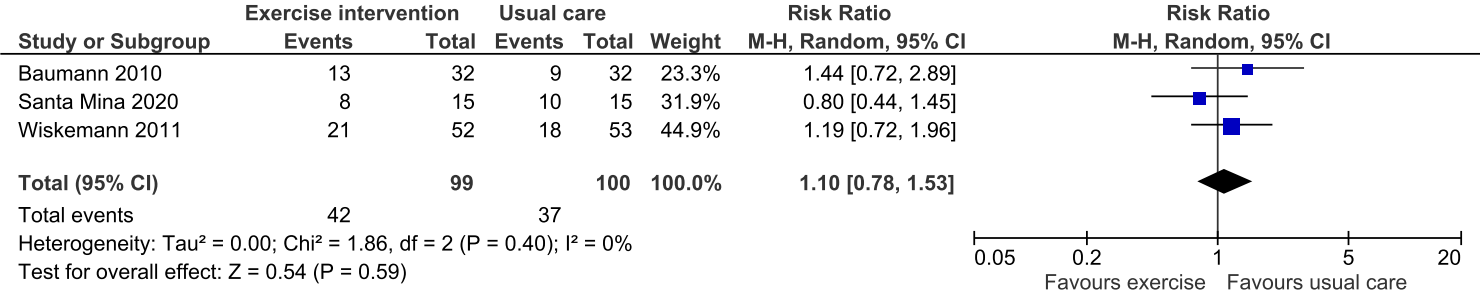

Supplement: Supplementary file 19 — Supplementary file19 (PDF 292 KB) [file 520_2021_6334_MOESM19_ESM.pdf]
